# Quantitative evaluation of China's smart aging healthcare policy under the background of silver economy development: based on PMC model

**DOI:** 10.3389/fpubh.2026.1791984

**Published:** 2026-04-16

**Authors:** Ying Guan, Yu Xiang, Yan Fu, Leyao Huang, Shiyu Su, Zaixiang Tan, Xinru Huang

**Affiliations:** 1 School of Management, Xuzhou Medical University, Xuzhou, China; 2 Institution of Chinese Health Modernization, Xuzhou Medical University, Xuzhou, Jiangsu, China; 3 The Affiliated Hospital of Xuzhou Medical University, Xuzhou, Jiangsu, China

**Keywords:** PMC-Index model, policy evaluation, policy quantification, policy tools, silver economy, smart aging care

## Abstract

**Background:**

Against the backdrop of deep integration between population aging and digital technologies, smart aging care has emerged as a pivotal approach to alleviating caregiving pressures, optimizing resource allocation for senior care, and driving high-quality development of the silver economy. As a top-level institutional design, the quality of smart aging care policies directly determines the effectiveness of older adult care services and the inclusiveness of digital technology applications in older adult care. Conducting a scientific and systematic quantitative evaluation of such policy documents is essential. This study aims to quantitatively evaluate the overall quality and regional heterogeneity of China's smart aging care policies, identify policy shortcomings, and propose optimization pathways for improvement.

**Methods:**

The PMC-Index model is the quantitative policy evaluation method that constructs a multi-dimensional indicator system and assigns equal weights to each indicator. Based on this, this study has constructed an evaluation index system for the PMC model of smart aging care policies. This study systematically reviewed 83 smart aging care policies issued by China's government from 2020 to 2025.10 representative policies were selected based on screening criteria that encompass regional coverage across eastern, central, and western regions, uneven economic development, reflection of aging disparities, and responsiveness to the digitalization process.The PMC index was calculated through integrated methods including text mining and multi-input-output tables, combined with visual analysis to evaluate policy structures and regional heterogeneity, thereby identifying optimization pathways.

**Results:**

The findings indicate that the overall quality of China's smart aging healthcare policies has reached the “excellent” level, characterized by clear objectives, comprehensive domain coverage, and rigorous textual logic, demonstrating the strong orientation toward technology empowerment and industrial synergy. However, challenges remain: insufficient policy continuity, imbalanced tool mixes, and underdeveloped safeguards. Moreover, policy quality exhibits regional heterogeneity, with eastern regions prioritizing innovation and industrial cultivation, whereas central and western regions focus more on basic service provision and inclusive implementation.

**Conclusion:**

This study employs the PMC-Index model to conduct the multidimensional quantitative assessment of China's smart aging care policies. This research provides the reference framework for optimizing and refining smart aging care policies in China, which may also offer insights for other countries and regions addressing population aging and fostering silver economy development. Ultimately, it aims to support the coordinated advancement and continuous improvement of global smart aging care policies.

## 1 Introduction

**Figure 1:**
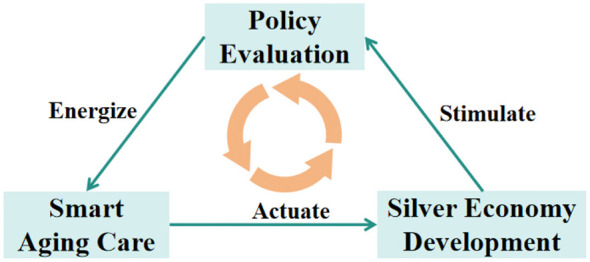
The Conceptual diagram of the relationship between policy evaluation, smart aging care and silver economy.

“Smart aging care”refers to the integration of older adult care resources utilizing information technologies such as the Internet of Things, cloud computing, big data, and artificial intelligence. It establishes a closed-loop service system covering home-based, community-based, and institutional settings through automated monitoring, early warning, and proactive intervention of older adult information (1, 2), achieved via deep integration of digital technologies and service resources (3). As the global population structure accelerates toward aging, developing technology-driven smart aging care models has become the strategic pathway to address caregiving pressures and enhance the quality of life for older adults. The term “silver economy” originated in Japan. During the 1970s, Japan experienced accelerated aging, with the older adult population steadily expanding and demand for older adult care-related consumption and services surging. There was an urgent need to establish a specialized concept to define economic activities targeting this demographic. “Silver” gradually became a distinctive symbol representing the older adult population (4). As the emerging economic modality for aging societies, the silver economy is a key lever to elevate older adult care service quality and foster new economic growth drivers. Smart aging care is the core vehicle for the silver economy's implementation and iterative upgrading, and their in-depth integration has become the pivotal trend in global aging governance. The World Health Organization's Global Report on Aging and Health emphasizes that advancing assistive technologies and building age-friendly environments are core strategies for maintaining and enhancing functional capacity among older populations (5). This concept was further expanded and reinforced in the United Nations' Decade of Action on Healthy Aging 2020–2030 global framework (6), jointly advocating for a practical and systematic pathway to achieve “healthy aging” through integrated approaches such as dynamic health monitoring, telemedicine support systems, and systematic age-friendly adaptations of physical and social environments. In recent years, smart aging care solutions have gained increasing global adoption. The integration of digital technologies has not only optimized the responsiveness of older adult care services but also expanded the scope of home-based and community-based care (7). Practices from 10 highly aged nations, including Japan and the EU, demonstrate that systematic smart aging care policies can effectively reduce long-term care costs (8, 9). The widespread adoption of smart monitoring and digital platforms enhances resource allocation efficiency by enabling preventive interventions and precise resource matching (10, 11). However, rapid development in smart aging care also brings challenges such as ethical norms gaps, inadequate technological adaptability, and weak digital equity (12, 13). In addressing the aforementioned challenges, different countries have developed differentiated policy approaches based on their respective institutional contexts. Japan has incorporated age-friendly products into the coverage scope by revising the Nursing Insurance Act, thereby driving technological innovation from the payment perspective and effectively unlocking the industrial potential of home-based smart aging care services (14). The EU, through its “Active and Assisted Living” programme, has established the fast-track pathway from research and development to market launch by providing cross-border funding and setting uniform technical standards (15, 16). Singapore is using AI-powered image screening technology to assist in the diagnosis of geriatric syndromes, significantly improving the accuracy and efficiency of chronic disease management (17). At the domestic implementation level, China exhibits characteristics of full-scenario integration and inclusive-driven development (18). By establishing a “collaborative service network” that encompasses intelligent monitoring, community healthcare, and home care, the country has achieved effective synergy between technological empowerment and resource decentralization (19). As institutional frameworks and top-level designs, the textual quality of smart aging care policies directly impacts the silver economy's standardizing and high-quality development. For instance, determining the overall trajectory of digital health service reform (DHSR) (20). Such institutional arrangements at the policy level, by regulating the effectiveness of technology application and the fairness of resource allocation, directly influence the level of inclusiveness and health equity objectives during the implementation of smart aging care services (21). Previous studies have addressed policy interpretation, policy instruments classification and regional case studies. However, the quantitative evaluation framework for comparisons across different levels and regions has not established, which makes more difficult to systematically identify structural shortcomings in policy documents and implementation (22). The PMC-Index model fills this research gap. This enables the objective quantification and visual analysis of policy text quality, providing a replicable, standardized framework for the fair comparison of policies across different levels and regions (23). The model also precisely decodes performance characteristics across policy dimensions, systematically identifies policy compatibility deficiencies and targeted optimization directions, thereby offering more scientific and actionable quantitative analysis tool for smart aging care policy evaluation (24). Therefore, this study adopts the perspective of silver economy, focuses on smart aging care, and employs the PMC-Index model to conduct a scientific evaluation of the quality of various smart aging care policies. Policy evaluation quantifies policy quality, thereby empowering the scientific design and precise implementation of smart aging care policies, and consequently promoting the more effective contribution of smart aging care to the silver economy. Meanwhile, the rapid growth of silver economy is constantly giving rise to new industrial demands, which in turn are driving the iterative optimization of smart aging care policies. This establishes a virtuous cycle logic where policy evaluation empowers smart aging care, smart aging care drives the silver economy, and the silver economy in turn nourishes policy evaluation. The conceptual diagram is shown in Figure 1. This research aims to advance the standardized development of smart aging care, strengthen policy support for technology application and service accessibility, and provide quantitative evidence for the iterative refinement of smart aging care policies. To facilitate analysis, this paper poses the following three questions: (1) What are the core dimensions influencing the effective implementation of smart aging care policies? (2) How can the PMC-Index model be used to quantitatively assess the quality and adaptability of smart aging care policies? (3) What optimization pathways exist to enhance the implementation effectiveness of smart aging care policies, and how can they be advanced?

## 2 Literature review

### 2.1 Research on AI in smart aging care

Artificial intelligence (AI) is a multidisciplinary approach integrating computer science and linguistics, designed to create machines capable of performing tasks that typically require human intelligence. Innovative older adult care products and services incorporating AI are recognized as pivotal methodologies for future geriatric medicine, with AI emerging as a core driving force in reshaping the global paradigm of intelligent older adult care services (7). As global academic and industry research deepens, AI has been extensively embedded in core scenarios such as health monitoring, precision care, and safety alerts (25). Leveraging its superior data processing and predictive analytics capabilities, AI has significantly enhanced the efficiency and precision of older adult care service delivery, effectively alleviating the global imbalance between resource supply and demand. Both the World Health Organization and the United Nations have highlighted in their reports that technological innovation is the key variable to enhance the quality of life for older adults and optimizing the effectiveness of older adult care services (5, 6). Currently, technological advancements in artificial intelligence for older adult care worldwide primarily focus on enhancing care quality and efficiency through auxiliary diagnosis, health monitoring, predictive analytics, and robotic support. Its specific application value has been demonstrated through practice in multiple countries. Norway developed a multi-modal sensing system based on mobile robots through the MECS (Multi-modal Elderly Care System) project, integrating depth, thermal imaging, and ultrasonic sensing technologies to achieve non-invasive real-time monitoring of older adult behavioral preferences and fall risks (26). Italy emphasizes the utilization of machine learning algorithms to predict personalized rehabilitation needs and integrates AI-driven devices for chronic pain management, significantly enhancing the physical functional independence and rehabilitation treatment adherence of older adult individuals (27). Singapore has developed a digital assessment tool for geriatric syndromes and utilized artificial intelligence technology to perform auxiliary diagnosis through retinal scanning, achieving interpretation accuracy comparable to that of physicians. Through this AI-based imaging screening and evaluation, the identification efficiency of chronic diseases in the older adult has been significantly improved, providing technical support for ensuring independent living at home and high-quality care (17). China has prioritized the development of smart home-based older adult care systems integrating the Internet of Things and artificial intelligence. Leveraging cloud-based integration and real-time big data processing (19), these systems have effectively achieved automated recognition of older adult individualsrioriti activities and early warning of potential risks, significantly enhancing the safety and quality of life for home-based older adult care. At the industrial level, AI technology is accelerating the collaborative development of the global smart aging care ecosystem (28). On one hand, leading tech companies are committed to developing full-scenario solutions (29), driving the cross-border transfer and universal application of technological achievements. On the other hand, the deep integration of AI with older adult care services has spawned diversified service paradigms (30), providing robust foundational support for model innovation. However, despite substantial progress in research and application, the deep integration of AI into the aging care sector is also facing severe challenges, which has become a focal point of current academic attention. From an ethical perspective, Dignum and Virginia pointed out that the deep involvement of AI exacerbates systemic risks such as data privacy breaches and algorithmic bias (31), urgently necessitating transnational collaboration to refine technological ethics and safety frameworks. From the viewpoint of digital inclusion, Ma et al. conducted an in-depth analysis of the technological adaptability barriers faced by the older populations (32). Research indicates that certain mobile applications fail to adequately accommodate the perceptual capabilities and cognitive load characteristics of older adults in their design logic. This disconnect in design directly limits technological accessibility and significantly reduces older users' willingness to adopt digital and intelligent products. Furthermore, the absence of unified technical standards and interoperability protocols within the industry creates “island effects” between devices and platforms, hindering the scalable development of services. In summary, while academia has explored dimensions such as risk prevention, technological adaptation, and standardization, research on regulatory frameworks for AI policies in smart aging care remains relatively lagging. This failure to keep pace with the practical demands of technological iteration and industrial upgrading underscores the urgent need for more forward-looking theoretical guidance and policy solutions in this field.

### 2.2 Research on the silver economy in smart aging care

Within the research framework of smart aging care, the silver economy has evolved from its initial conceptual form into a comprehensive system of products and services driven by digital intelligence. This system addresses the entire life cycle of the older populations and encompasses related economic activities. The silver economy is a comprehensive lifecycle initiative spanning both pre-retirement preparation and actual aging phases. Its core logic lies in proactively addressing demographic transition challenges through tailored product and service provision, constituting a highly integrated economic model (33). As the core economic vehicle for implementing smart aging care, the connotation of the silver economy can be defined from both supply and demand perspectives. On the supply side, it leverages foundational technological innovations like artificial intelligence, the Internet of Things, and big data to transform traditional wellness industries into high-value-added, digital-intelligence industrial clusters encompassing smart monitoring devices, telemedicine terminals, and intelligent care systems (34). On the demand side, it not only focuses on the multi-level material and spiritual consumption of the older populations but also emphasizes the economic contribution of technological intervention to enhancing the quality of life and social participation of older adults (35). Its economic benefits possess dual attributes: they are manifested both in the economies of scale unleashed by accurately matching the domestic demand of the older populations, and in the macro-level social benefits achieved by substituting technology for labor, leading to the structural optimization of the total societal cost of long-term care. From the global perspective, although countries differ in their terminologies, such as “silver economy,” “longevity economy,” or “older adult care industry,” their logical starting point profoundly aligns with the industrial paradigm shift driven by demographic transition. As argued by Coughlin, this economic form is essentially the fastest-growing and most promising misunderstood market globally. It is not merely the response to aging societies but a core engine driving cross-industry technological innovation and market restructuring (36). This transformative logic transcends the narrow confines of traditional older adult care services, converting demographic pressures into endogenous forces that drive digital intelligence upgrades across industrial chains and reshape consumption patterns. Consequently, a high degree of consensus on “demographic transition-induced industrial innovation” has been achieved globally. China's silver economy industry mainly relies on policy encouragement and promotion (37). Germany leverages family support policies and long-term care insurance systems to enhance technological applications and inter-generational mutual assistance in home-based care, while promoting inter-generational digital inclusion through community spaces such as “multi-generational houses.” (38). The United States promotes innovative smart aging care through market-oriented approaches, such as Cariloop (a comprehensive nursing support platform), which integrates digital nursing resources to alleviate caregiving pressures for the “sandwich generation” and fill gaps in public services (38). However, existing research indicates that the global silver economy, as related to smart aging care, still faces common deficiencies such as the over-principled nature of policies and insufficiently precise support for critical links in the industrial chain. From the perspective of global industrial division of labor, significant regional development imbalances exist within the smart aging care sector, with the global silver economy exhibiting a pronounced dual structure of technology and capital. A relevant report by the United Nations confirms the existence of a significant “digital divide” in aging-related technologies between developed and developing countries, with the latter generally facing the dual constraints of high costs associated with introducing core technologies and insufficient competitiveness in their domestic industrial chains (39). This misalignment of capital and technology makes expensive digital aging care solutions unaffordable for developing countries. However, empirical research on transnational industrial collaboration and global market linkage logic remains scarce, necessitating deeper exploration in future studies to promote balanced development of the global smart aging care industry. Drawing on international precedents, Japan amended its Long-Term Care Insurance Act to include aging-friendly products within coverage, effectively incentivizing technological innovation at the production end through payment mechanisms. Quantitative research by Nishino substantiates a marked deinstitutionalization trend in Japan's macro-level supply-demand structure, concurrent with the societal shift toward an “aging in place” care paradigm (14). Data indicates a sustained rise in demand ratios for community multifunctional care and home living facilities, while the target for traditional large-scale institutions has declined. This structural adjustment in supply and demand, mediated through the insurance payment system, has directly unlocked the market potential for home-based aging-adaptation products. It has mandatorily steered industrial research and development away from standalone hardware toward integrated smart aging care solutions that unify community and home settings, thereby providing an institutional paradigm for the precise matching of supply and demand within the silver economy. The EU's “Ambient Assisted Living” (AAL) programme has established a fast-track pathway from research and development to market through the allocation of transnational funding, public-private partnerships (PPP) and harmonized technical access standards. As demonstrated by the case study of Bajenaru et al. (16) this systematic approach not only accelerates the adoption of smart assistive technologies in the aging care market but also ensures high alignment between technological development and the actual needs of older adults through standardized satisfaction assessment systems. Adopting such models can help China break the path dependence in the translation of smart aging care outcomes and enhance industrial synergy. By leveraging these cross-border integration approaches, China should focus on optimizing resource allocation. While providing targeted support to critical links in the industrial chain, it should actively lead or participate in establishing global standards for age-friendly technologies, thereby establishing a comprehensive response mechanism that bridges “basic research to industrial transformation” and fosters deep coupling between scientific research and market incubation. Furthermore, fiscal and taxation levers should be employed to integrate high-frequency smart aging care products into the social security or inclusive financial systems, which would unlock the synergistic potential of the silver economy to drive industrial upgrading and enhance the wellbeing of the aging population at a profound level.

### 2.3 Practical research on smart healthcare services for older adults

Within the macro landscape of smart aging care, intelligent medical services have emerged as the most dynamic subsector, whose core value lies in driving the paradigm shift of older adult health management from “passive response” to “active prevention” (40). Grounded in global academic perspectives and industrial practice observations, this field has preliminarily established a digital-intelligence service system covering the entire life cycle, exhibiting distinct characteristics of policy-driven development and technology-enabled advancement. Different countries and regions have developed distinctive approaches based on their own demographic structures, cultural backgrounds and technological foundations. The EU's smart aging initiatives focus on digital literacy and Ambient Assisted Living (AAL), integrating sensors, the Internet of Things and artificial intelligence algorithms at a technical level to build home monitoring platforms that enable precise risk alerts, including fall detection, physiological monitoring and behavioral analysis. At the same time, they use the Digital Skills Index (DSI) to dynamically assess the digital literacy of older adults, and work to bridge the digital divide through personalized training and accessible interaction design (15). Under the framework of its “Vision 2030” national strategy, Saudi Arabia is focusing on the intelligent transformation of healthcare, centered on AI-driven conversational agents. By integrating natural language processing (NLP) with electronic health record (EHR) systems, the technology enables a comprehensive suite of services, including 24/7 medical consultations, personalized prescription management and appointment scheduling (41). Examining international institutional evolution, the implementation of telemedicine policies has laid the legal and regulatory groundwork for intelligent transformation. For instance, building upon the structured Prescribing Safety framework proposed by the American Geriatrics Society (AGS), the integration of AI technology has enabled deep convergence between clinical decision support and remote data flows, thereby triggering the large-scale application and standardized transformation of remote diagnosis and treatment models globally (42). In addition, South Korea and the United Kingdom have focused on large-scale construction of intelligent age-friendly housing, establishing an integrated model combining housing, services, and technology (43). Concurrently, smart aging care initiatives in China are characterized by comprehensive integration across all scenarios and a focus on universal access, having established a multi-dimensional ecosystem that encompasses smart wearable monitoring, smart home sensing, emotional support robots and AI-assisted diagnosis and treatment (18). In addition, China is also focusing on the coordinated development of its older adult care service system. Pilot cities have adopted an integrated linkage model involving the deployment of smart devices, the stationing of community healthcare professionals, and the engagement of family caregivers. This model, supported by the artificial intelligence data platform, has enabled the dynamic tracking of health indicators among the older populations and the precise allocation of regional medical resources (19). This “collaborative service network,” which balances technological breadth and humanistic considerations, provides a resilient Chinese paradigm for older adult care during societal transition. Indeed, within the rapidly expanding global context, the equity and standardization of intelligent medical services remain critical challenges. Wubineh further noted that addressing such technical bottlenecks urgently requires building multi-center, standardized datasets to enhance model generalization capabilities and service stability (44). This shift from localized optimization to standardized collaboration is essential for ensuring the safety and reliability of smart healthcare decisions across diverse environments. Meanwhile, regional development disparities remain a core obstacle to achieving universal service coverage. Systematic advancement of smart healthcare necessitates the downward allocation of technological resources to rural and underdeveloped areas to bridge the “digital divide” caused by infrastructural differences (45). Only through standardized development and deep coverage of high-need scenarios can the high-quality and equitable delivery of intelligent medical services be guaranteed across varying socioeconomic contexts. While China is continuously improving service coverage in rural and remote areas through resource allocation, empirical research reminds us that future efforts must deepen the focus on high-need scenarios in underdeveloped regions, ensuring that technological iterations can accurately reach the furthest ends of society (12). This global exploration, expanding from individual points to broader coverage, signals that smart healthcare services for older adults are entering a critical phase of transition from quantitative to qualitative advancement. As technological bottlenecks are overcome and resource allocation mechanisms continuously optimized, this field will gradually resolve existing structural contradictions, ultimately achieving balanced, high-quality development that encompasses all populations and spatial dimensions.

In summary, existing international and domestic research has yielded foundational insights into the technological application of smart aging care, its related silver economy vehicles, and intelligent medical service practices, which collectively reveal both the common trends in global smart aging care development and the differentiated characteristics among nations. Against this backdrop, this paper adopts an international perspective and focuses specifically on China's policies and practices, introducing the PMC-Index model to conduct a systematic quantitative analysis of smart aging care policy texts. The study aims to construct a multidimensional evaluation system to precisely identify the quality characteristics and core compatibility issues of China's smart aging care policies. By integrating international experience with China's national conditions, it proposes scientifically feasible policy optimization pathways. This provides robust evidence for advancing the high-quality development of smart aging care in China, strengthening policy support for technology implementation and universal service access, and offering Chinese insights for refining global smart aging care policies.

## 3 Research design

### 3.1 Data sources and processing

The concept of smart aging care in China was first proposed by the National Office on Aging in 2021 and subsequently incorporated into national development plans in the following years. In 2015, the State Council issued the “Guiding Opinions on Actively Promoting the ‘Internet Plus' Initiative,” which explicitly proposed the concept of “smart health and aging care.” In 2017, the Ministry of Industry and Information Technology, the Ministry of Civil Affairs, and the National Health Commission jointly issued the “Action Plan for the Development of the Smart Health and Aging Care Industry (2017–2020),” marking the formal elevation of smart aging care to a national strategy. With the advancement of artificial intelligence and internet technologies in recent years, smart aging care has entered a new phase of development (46). Therefore, this study collected policy documents related to smart aging care in China from July 2020 to January 2025, using the keywords “smart and aging care services,” “Internet Plus and aging care,” “silver economy and aging care,” “artificial intelligence aging care products,” and “digital technology for aging-friendly adaptation” for our search. The policy documents are primarily sourced from official platforms such as those of the central government and provincial and municipal-level people's governments.

Inclusion Criteria: (1) The policy documents must be directly related to smart aging care, explicitly stipulating or reflecting relevant content. (2) The types of policies included in the study comprise: planning policy, guideline policy, Implementation policy, Measures policy, Notification-type policy. (3) Selected policies must be issued by national-level agencies such as the State Council, the Ministry of Industry and Information Technology, and the Ministry of Civil Affairs; provincial and municipal government offices; or municipal government offices and civil affairs bureaus. This three-tiered selection approach is adopted to better understand the current landscape of smart aging care policies in a stratified manner, ensuring the selected texts are more targeted, instructive, and authoritative. Exclusion criteria: (1) Policies that have been repealed; (2) Documents such as policy interpretations or news media reports; (3) Documents duplicating content already included in the policy text repository. Following careful reading and screening, as well as review by 1–2 experts in the fields of older adult care and public policy, 10 policies that did not meet the criteria were excluded, resulting in the final inclusion of 83 policy documents issued between 2020 and 2025.

### 3.2 Construction of the PMC-Index model

**Figure 2:**
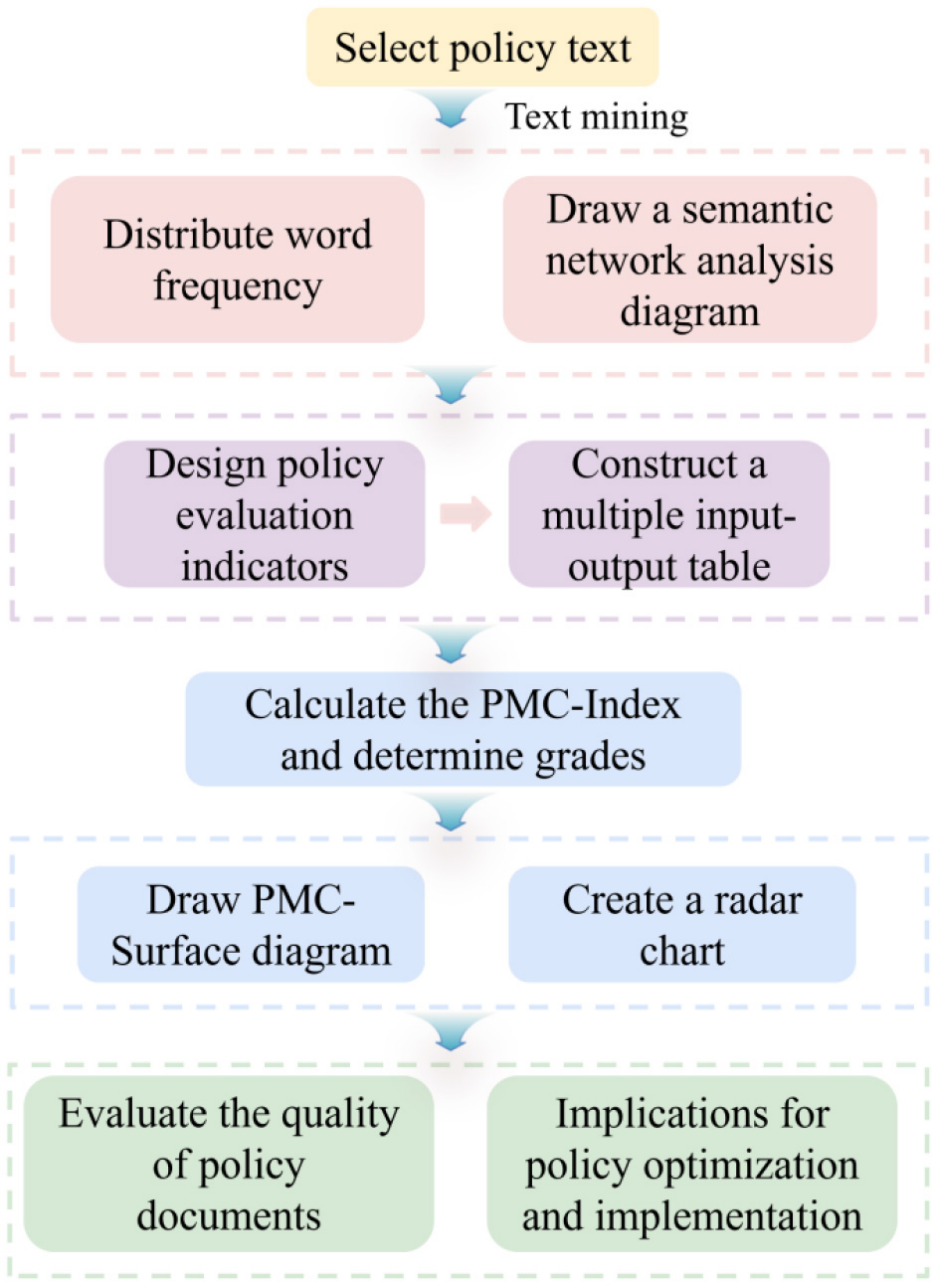
Flowchart.

The PMC-Index model originates from the “Omnia Mobilis” hypothesis proposed by Estrada (23), which posits that all elements within a policy system are interconnected and undergo continuous change, rendering every variable of significant importance. Based on this theory, the PMC-Index model conducts systematic, quantitative policy evaluations by constructing multiple primary variables and their corresponding secondary variables. In practical applications, models typically establish 9 to 10 primary indicators, each comprising multiple secondary indicators with equal weighting and standardized scoring criteria (24). This approach aligns with the core theoretical framework of the model, as no element in the policy system holds absolute primacy. Equal weighting effectively mitigates subjective biases arising from manual weighting decisions, ensuring the objectivity and scientific validity of evaluation outcomes. The indicators are assigned binary values to minimize subjective bias. Following the structured analysis of policy texts, the assignment of scores to indicators and the aggregation of data, the PMC-Index is ultimately calculated (23). To enhance evaluation clarity, PMC-Surface visualizes index distributions as three-dimensional surfaces, making policy strengths and weaknesses immediately apparent. Combining PMC-Index with PMC-Surface enables comparisons between individual or multiple policies, providing quantitative evidence and graphical support for policy optimization. The specific workflow is illustrated in Figure 2.

### 3.3 Word frequency analysis and variable design

#### 3.3.1 Word frequency analysis

This study utilized the ROSTCM6 text mining software to compile the selected 83 policy texts into TXT format files, which were then subjected to in-depth analysis and word frequency statistics. The preprocessing steps for text mining included: (1) Merging 83 policy texts on smart aging care into a single file and importing it into ROSTCM 6.0 for word segmentation processing. (2) Text cleaning, which involved removing irrelevant metadata (such as “issuing department” and “document number”) and subsequently conducting word segmentation; (3) Building upon the standard stop word list built into ROSTCM 6.0 by adding a customized list of domain-specific terms relevant to this research topic, followed by stemming and retaining the top 50 high-frequency words; (4) Eliminating irrelevant words by removing pronouns, prepositions, and other terms with minimal semantic weight, such as “should,” “then,” and “promote.” Simultaneously, merge duplicate standard terms.

**Figure 3:**
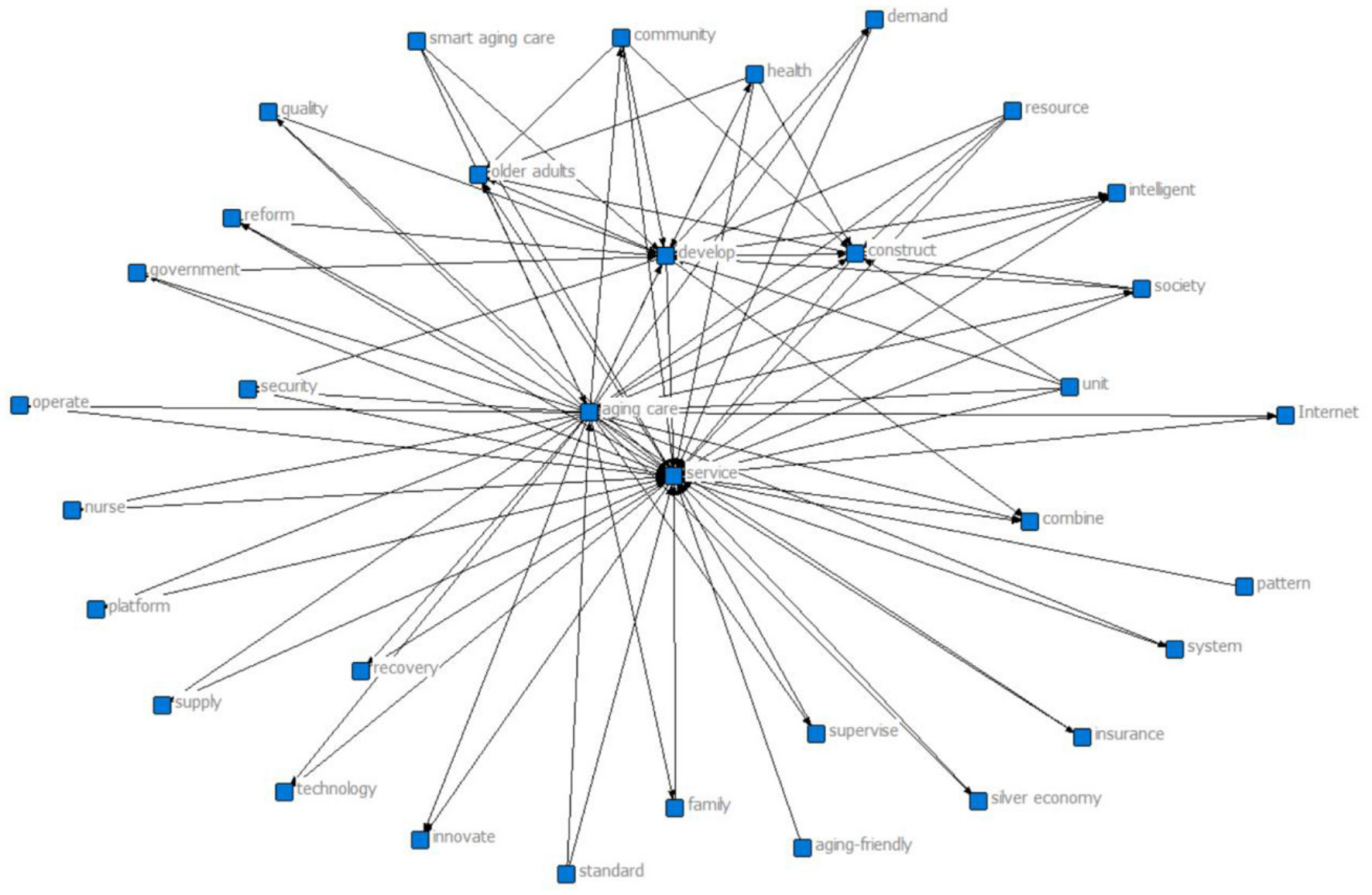
Semantic network diagram of high-frequency co-occurring terms in smart aging care policy texts.

Following the preprocessing, the filtered 40 high-frequency terms were compiled into a high-frequency word list (Table 1). These terms were then mapped onto the initial evaluation indicators. For instance, high-frequency terms such as “service” (appearing 9,856 times) and “aging care” (appearing 9,609 times) were incorporated as variable criteria in X_6_ (Policy Content) and X_7_ (Policy Objectives). Subsequently, based on the extracted high-frequency keywords, we utilized ROSTCM 6.0 software to construct a semantic network diagram, as shown in Figure 3. Within this semantic network, the core nodes located at the center of the diagram include “service,” “aging care,” “older adults,” “develop,” “government,” “health,” and “wellness,” with the remaining keywords distributed around these central concepts. The number of connections between keywords reflects the closeness of their relationships and the strength of their associations. Notably, keywords such as “service,” “government,” “aging care,” and “health” highlight key areas requiring further strengthened connections within the policy framework. Therefore, when constructing a multi-input-output policy variable analysis, priority should be given to their deep integration and reinforcement.

**Table 1:** High-frequency words statistics in policy texts.

Sequence	High frequency words	Frequency	Sequence	High frequency words	Frequency
1	Service	9,856	21	Application	588
2	Aging care	9,509	22	Quality	584
3	Older	4,524	23	Insurance	580
4	Develop	2,540	24	Care	572
5	Construct	1,910	25	Recovery	547
6	Society	1,496	26	Age-friendly	541
7	Health	1,460	27	Platform	537
8	Community	1,433	28	Sound	532
9	Nursing	1,052	29	Innovate	517
10	Government	1,038	30	System	516
11	Civil affairs	1,034	31	Renovation	513
12	Security	887	32	Disability	495
13	Establish	833	33	Field	479
14	Standard	817	34	Operation	462
15	Economy	751	35	Silver economy	459
16	Intellectual	739	36	Data	453
17	Resource	737	37	Smart aging care	448
18	Technology	672	38	Education	444
19	Supervise	653	39	Supply	438
20	Demand	620	40	Reform	429

#### 3.3.2 Variable design

Based on the aforementioned theoretical foundations, this study systematically incorporates the original indicator framework proposed by Estrada (23), along with existing research findings by Yang et al. (47) and Guo et al. (48), while integrating the distinctive characteristics of smart aging care policies to conduct indicator selection and optimization. First, adhering to public policy evaluation standards and the original design logic of the PMC model, we conducted an initial screening to identify core dimensions that align with policy essence and cover the entire policy process. Indicators that were overly broad, lacked statistical significance, or had poor compatibility with smart aging care policies were excluded. This approach enabled precise definition of the connotation of primary variables. Secondly, by integrating the text mining results, we conducted an in-depth analysis of the high-frequency elements, core logic, and key influencing factors in the policy text on smart aging care. We then selectively identified secondary indicators that align with policy scenarios, are supported by empirical evidence, and fit China's economic and implementation contexts. This ensures that the secondary indicators accurately reflect the core essence of the primary variables and are compatible with the actual implementation of the policy. After multiple rounds of validation, screening, and expert consultation optimization, 10 primary variables and 43 secondary variables were ultimately identified to construct a PMC-Index model tailored to China's smart aging care policies. The specific variable settings are detailed in Table 2. Among them, the 10 primary variables include policy nature, policy timeliness, policy domain, policy object, policy tool, policy content, policy objectives, policy measures, policy evaluation, and policy openness. Given that all policies included in the study possess statutory transparency requirements under the policy openness (X_10_) dimension, quantitative evaluation can be completed without further subdivision into secondary dimensions. Therefore, no secondary indicators were established for this variable.

**Table 2:** Evaluation indicator system for smart aging care policies.

Primary variables	Secondary variables	Meaning of secondary variable
Policy Nature (X_1_)	X_11_Prediction	Whether the policy contains predictive content: Yes = 1, No = 0
	X_12_Guidance	Whether the policy contains guiding content: Yes = 1, No = 0
	X_13_ Support	Whether the policy contains supporting content: Yes = 1, No = 0
	X_14_Regulation	Whether the policy contains regulatory content: Yes = 1, No = 0
	X_15_Description	Whether the policy contains descriptive content: Yes = 1, No = 0
	X_16_Diagnosis	Whether the policy contains diagnostic content: Yes = 1, No = 0
Policy Timeliness (X_2_)	X_21_Short–term	Whether the policy includes the content within 3 years: Yes = 1, No = 0
	X_22_Medium–term	Whether the policy includes the content of 3–5 years: Yes = 1, No = 0
	X_23_Long–term	Whether the policy includes the content of more than 5 years: Yes = 1, No = 0
Policy Domain (X_3_)	X_31_Economy	Whether the policy involves the economic sector: Yes = 1, No = 0
	X_32_Healthcare	Whether the policy involves the healthcare sector: Yes = 1, No = 0
	X_33_Technology	Whether the policy involves the technology sector: Yes = 1, No = 0
	X_34_Social services	Whether the policy involves the social services sector: Yes = 1, No = 0
Policy Object (X_4_)	X_41_Government	Whether the policy object involves the government: Yes = 1, No = 0
	X_42_Enterprise	Whether the policy object involves the enterprise: Yes = 1, No = 0
	X_43_Research institution	Whether the policy object involves the research institution: Yes = 1, No = 0
	X_44_Community	Whether the policy object involves the community: Yes = 1, No = 0
	X_45_Family	Whether the policy object involves the family: Yes = 1, No = 0
Policy Tool (X_5_)	X_51_Mandatory	Whether the policy is mandatory: Yes = 1, No = 0
	X_52_Incentive–based	Whether the policy is Incentive–based: Yes = 1, No = 0
	X_53_Innovative	Whether the policy is innovative: Yes = 1, No = 0
	X_54_Market–oriented	Whether the policy is market–oriented: Yes = 1, No = 0
	X_55_Collaborative	Whether the policy is collaborative: Yes = 1, No = 0
Policy Content (X_6_)	X_61_Smart health and aging care	Whether the content of the policy involves smart health and aging care: Yes = 1, No = 0
	X_62_Older adult care service scenario applications	Whether the content of the policy involves older adult care service scenario applications: Yes = 1, No = 0
	X_63_Age-friendly renovations	Whether the content of the policy involves age-friendly renovations: Yes = 1, No = 0
	X_64_Digital platform development	Whether the content of the policy involves digital platform development: Yes = 1, No = 0
	X_65_Older adult education and training	Whether the content of the policy involves older adult education and training: Yes = 1, No = 0
	X_66_Insurance for older adults	Whether the content of the policy involves insurance for older adults: Yes = 1, No = 0
Policy Objectives (X_7_)	X_71_Product innovation	Whether the policy achieves product innovation: Yes = 1, No = 0
	X_72_Technology application	Whether the policy involves technology application: Yes = 1, No = 0
	X_73_Model promotion	Whether the policy promotes model dissemination: Yes = 1, No = 0
	X_74_Standard development	Whether the policy establishes standards: Yes = 1, No = 0
	X_75_Improving the quality of older adult care services	Whether the policy enhances the quality of older adult care services: Yes = 1, No = 0
	X_76_Developing the silver economy industry	Whether the policy fosters the development of the silver economy industry: Yes = 1, No = 0
Policy Measures (X_8_)	X_81_Legal safeguards	Whether the policy includes legal safeguards: Yes = 1, No = 0
	X_82_Funding support	Whether the policy includes funding support: Yes = 1, No = 0
	X_83_Talent development	Whether the policy includes talent development: Yes = 1, No = 0
	X_84_Model promotion	Whether the policy includes model promotion: Yes = 1, No = 0
Policy Evaluation (X_9_)	X_91_Sufficient basis	Whether the policy basis is sufficient: Yes = 1, No = 0
	X_92_Clear objectives	Whether the policy objectives are clear: Yes = 1, No = 0
	X_93_Detailed program	Whether the policy program is detailed: Yes = 1, No = 0
	X_94_Scientific planning	Whether the policy planning is scientific: Yes = 1, No = 0
Policy openness (X_10_)	/	/

### 3.4 Constructing a multi-input–output table

To quantitatively evaluate future smart aging care policies, this study has constructed a multi-input-output matrix framework. This framework can process and store large volumes of relevant policy data, measuring variables within policy texts across multiple dimensions. Following the construction principles of the PMC-Index model, all primary and secondary variables are assigned equal weights. The values of secondary variables follow a [0, 1] distribution, taking either 0 or 1. A value of 1 indicates that the policy content includes or addresses the relevant variable, while 0 signifies no relevance. This approach is based on the characteristic of multi-dimensional measurement of policy content, providing foundational data support for the subsequent calculation of the PMC-Index, as illustrated in Table 3.

**Table 3:** Multi-input-output table.

Primary variables	Secondary variables
X_1_	X_11_, X_12_, X_13_, X_14_, X_15_, X_16_
X_2_	X_21_, X_22_, X_23_
X_3_	X_31_, X_32_, X_33_, X_34_
X_4_	X_41_, X_42_, X_43_, X_44_, X_45_
X_5_	X_51_, X_52_, X_53_, X_54_, X_55_
X_6_	X_61_, X_62_, X_63_, X_64_, X_65_, X_66_
X_7_	X_71_, X_72_, X_73_, X_74_, X_75_, X_76_
X_8_	X_81_, X_82_, X_83_, X_84_
X_9_	X_91_, X_92_, X_93_, X_94_
X_10_	/

### 3.5 PMC-index calculation and evaluation grade classification

The calculation method for the PMC-Index model is as follows: First, assign relevant weights to all secondary indicators in the multi-input-output table. According to Equations 1 and 2, if the smart aging care policy within a specific policy text contains a given secondary indicator variable, the parameter value is assigned as 1; otherwise, it is assigned as 0. For example, the secondary variable “Digital Platform Development” corresponds to the provisions in the Shanghai policy document regarding “the development of a public service platform for research, development and testing” and “the development of a comprehensive platform for basic older adult care services”. Therefore, it is assigned a value of 1. Furthermore, the policy document issued by Zhejiang Province states that “the ‘Zhe li Ban' app will enhance the level of age-friendly and accessible modifications to its internet applications”. As this relates to the meaning of this indicator, it is also assigned a value of 1.Next, the value of each primary variable is calculated according to Equation 3. Finally, combine the scores of all primary variables using Equation 4 to calculate the overall PMC-Index (49).


$$\begin{array}{l} X\sim N\left[0,1\right] \end{array}$$ (1)

$$\begin{array}{l} X=\left\{XR:\left[0,1\right]\right\} \end{array}$$ (2)

$$\begin{array}{l} X_{i}\left(\overset{n}{\underset{j=1}{\sum }}\frac{X_{ij}}{T\left(X_{ij}\right)}\right),i=1,2,3,n \end{array}$$ (3)

$$\begin{array}{l} PMC=\left[\begin{array}{c} X_{1}\left[\overset{6}{\underset{j=1}{\sum }}\frac{X_{1j}}{6}\right]+X_{2}\left[\overset{3}{\underset{j=1}{\sum }}\frac{X_{2j}}{3}\right]+\\ X_{3}\left[\overset{4}{\underset{j=1}{\sum }}\frac{X_{3j}}{4}\right]+X_{4}\left[\overset{5}{\underset{j=1}{\sum }}\frac{X_{4j}}{5}\right]+\\ X_{5}\left[\overset{5}{\underset{j=1}{\sum }}\frac{X_{5j}}{5}\right]+X_{6}\left[\overset{6}{\underset{j=1}{\sum }}\frac{X_{6j}}{6}\right]+\\ X_{7}\left[\overset{6}{\underset{j=1}{\sum }}\frac{X_{7j}}{6}\right]+X_{8}\left[\overset{4}{\underset{j=1}{\sum }}\frac{X_{8j}}{4}\right]+\\ X_{9}\left[\overset{4}{\underset{j=1}{\sum }}\frac{X_{9j}}{4}\right]+X_{10} \end{array}\right] \end{array}$$ (4)

i is a primary variable; j is a secondary variable; n is the number of secondary variables corresponding to primary variables i and j, where *n* = 1, 2, 3, 4, ….

### 3.6 Construction of PMC-surface diagrams

To present the evaluation results of the PMC-Index for smart aging care policies more intuitively and clearly, this study utilizes MATLAB software to visualize the PMC matrix and construct the PMC-Surface. The PMC-Surface is a 3D surface plot of a 3 × 3 matrix, which consists of the remaining nine first-level variables after excluding the X_10_ “Policy Openness” variable (24). The PMC-Surface uses convex and concave shapes to represent score levels, thereby illustrating the strengths and weaknesses of policies across different dimensions. Convex areas indicate higher levels, while concave areas represent lower scores, signifying dimensions with weaker policy performance. The values of the PMC matrix are calculated using Equation 5, and the PMC-Surface is plotted accordingly, providing a visual representation to support policy evaluation.


$$\begin{array}{l} PMC-Surface\;=\left[\begin{array}{c} X_{1}\;X_{2}\;X_{3}\\ X_{4}\;X_{5}\;X_{6}\\ X_{7}\;X_{8}\;X_{9} \end{array}\right] \end{array}$$ (5)

## 4 Quantitative analysis of smart aging care policies

### 4.1 Selection of representative policies

Based on an initial analysis of 83 policy documents on smart aging care and incorporating insights from domain experts, this study adheres to the principles of scientific rigor, representativeness, and hierarchical structure. We conducted in-depth quantitative evaluations by stratified and categorized sampling of 10 policy documents. The specific criteria for sample selection are outlined below:

1. Taking into account regional distribution and economic development level: The sample covers three major economic regions–eastern, central, and western China–while balancing developed and underdeveloped areas. At the provincial and municipal levels, the selected regions include developed eastern areas (Zhejiang Province and Shanghai), western hub regions (Chongqing), and central regions (Shanxi Province). The municipal-level selection includes developed eastern prefecture-level cities (Suzhou) and central provincial capitals (Wuhan). Comprehensive reflection of policy layout disparities in smart aging care under different levels of economic development.
2. Correlation between Aging Degree and Digital Infrastructure: The selected regional samples exhibit gradient differences in population aging levels and digital infrastructure development, accurately reflecting local governments' policy priorities and responsiveness to smart aging care under varying pension demands and digital empowerment conditions, ensuring the generalizability of the sample study.
3. Covering multiple policy levels and time efficiency: The sample covers four major policy levels: national, provincial, municipal directly under the central government, and city-level, balancing the implementation of national macro policies with detailed local implementation rules. Simultaneously, select currently effective policies issued within the past 5 years, excluding repealed or expired documents, to align with the current development trends of the smart aging care industry, ensuring the timeliness and practical guidance value of policy evaluation.

Through rigorous screening based on the established criteria, the final selection of 10 policy samples demonstrates geographical representativeness, hierarchical differentiation, and targeted content. These samples comprehensively reflect the core characteristics and implementation effectiveness of smart aging care policies across China's diverse regions and developmental contexts, thereby establishing a robust data foundation for subsequent quantitative PMC assessments.

In terms of release dates, the policy texts span from 2020 to 2025. They encompass both early solutions addressing “the difficulties faced by older adults in using smart technologies” and cutting-edge topics intensively introduced in recent years, such as “age-friendly digital technologies” and the “silver economy.” This reflects the continuous evolution of policy themes alongside socioeconomic development.

In terms of content orientation, national-level policies (P1–P4) emphasize top-level design, system construction, and cross-departmental coordination. Provincial and municipal policies (P5–P8), while implementing national requirements, demonstrate greater regional characteristics and innovative pilot initiatives. Municipal-level policies (P9–P10) focus more on operational feasibility and concrete implementation pathways, reflecting grassroots explorations in resource integration and service delivery.

Consequently, the 10 policies selected for this study not only exhibit systematic and comparative characteristics in terms of release dates, policy levels, and geographic distribution, but also cover multiple dimensions from macro-level strategies to micro-level implementation. This provides a comprehensive and structured textual foundation for subsequent policy text analysis and evaluation. The specific policy list is shown in Table 4.

**Table 4:** Representative policies for smart aging care.

Item	Policy name	Issuing authority	Date of release
1	Implementation plan for effectively addressing the challenges older adult individuals face in using smart technologies	General office of the state council	15 November 2020
2	Action plan for the development of the smart health and aging care industry (2021–2025)	Ministry of civil affairs, ministry of industry and information technology	20 October 2021
3	Action plan for promoting high-quality development of digital technologies suitable for older adults	\Ministry of industry and information technology	19 December 2023
4	Several measures to further promote older adult care service consumption and enhance the quality of life for older adults	Ministry of civil affairs, national development and reform commission, etc.	31 October 2024
5	Shaanxi province work plan for promoting high-quality development of digital technologies adapted for the older adult	Shaanxi provincial health commission, Shaanxi provincial department of industry and information technology, etc.	17 May 2024
6	Shanghai action plan for advancing the development of older adult care technology innovation (2024–2027)	General office of the shanghai municipal people's government	13 June 2024
7	Zhejiang province special action plan for promoting high-quality development of digital technologies for the older adult: “Zhejiang age-friendly”	Zhejiang provincial development and reform commission, Zhejiang provincial health commission, etc.	12 April 2024
8	Implementation plan for promoting the silver economy and enhancing the wellbeing of older adults in Chongqing	Chongqing municipal commission of economy and information technology, Chongqing municipal health commission, etc.	21 November 2024
9	Implementation plan for the Wuhan artificial intelligence older adult care social experiment	Wuhan municipal bureau of civil affairs	6 July 2020
10	Several measures to promote the high-quality development of Suzhou's silver economy	Suzhou municipal people's government	31 December 2024

### 4.2 PMC-Index of 10 representative policies

Based on the calculation process of the constructed PMC-Index model described above, text mining and content analysis were employed to assign values to the 10 smart aging care policy texts, determine the scores for secondary indicators, and construct a multi-input-output table, as shown in Table 5. Subsequently, calculations were performed on the assigned values in the multi-input-output table using Equation 4 to derive the PMC-Index and rank the final scores. The results are presented in Table 6.

**Table 5:** Multi-input–output table of 10 representative policies.

Primary variables	Secondary variables	P1	P2	P3	P4	P5	P6	P7	P8	P9	P10
X_1_	X_11_	1	1	1	0	1	1	1	1	1	1
	X_12_	1	1	1	1	1	1	1	1	1	1
	X_13_	1	1	1	1	1	1	1	1	1	1
	X_14_	1	1	1	1	1	1	1	1	1	1
	X_15_	1	1	1	1	1	1	1	1	1	1
	X_16_	1	1	1	1	1	1	1	1	1	1
X_2_	X_21_	1	0	1	0	1	1	0	0	0	0
	X_22_	0	1	0	0	0	0	1	1	1	1
	X_23_	1	1	1	1	1	1	1	0	1	0
X_3_	X_31_	1	1	1	1	1	1	1	1	0	1
	X_32_	1	1	1	1	1	1	1	1	1	1
	X_33_	1	1	1	1	1	1	1	1	1	1
	X_34_	1	1	1	1	1	1	1	1	1	1
X_4_	X_41_	1	1	1	1	1	1	1	1	1	1
	X_42_	1	1	1	1	1	1	1	1	1	1
	X_43_	0	1	1	0	0	1	1	1	1	1
	X_44_	1	1	0	1	0	0	0	1	1	1
	X_45_	1	1	0	1	0	0	0	0	1	1
X_5_	X_51_	1	0	0	1	0	0	1	0	0	0
	X_52_	1	1	1	1	1	1	1	1	1	1
	X_53_	1	1	1	1	1	1	1	1	1	1
	X_54_	0	1	1	1	1	1	0	1	1	1
	X_55_	1	1	1	1	1	1	1	1	1	1
X_6_	X_61_	1	1	1	1	1	1	1	1	1	1
	X_62_	1	1	1	1	1	0	1	1	1	1
	X_63_	1	1	1	1	1	1	1	1	1	1
	X_64_	1	1	1	1	1	1	0	1	1	1
	X_65_	1	1	0	1	0	0	0	1	0	0
	X_66_	0	0	0	1	0	1	0	1	0	1
X_7_	X_71_	1	1	1	1	1	1	1	1	1	1
	X_72_	1	1	1	1	1	1	1	1	1	1
	X_73_	1	1	1	1	1	0	0	1	1	0
	X_74_	1	1	1	1	1	1	1	1	0	1
	X_75_	1	1	1	1	1	1	1	1	1	1
	X_76_	1	1	1	1	1	1	1	1	0	1
X_8_	X_81_	1	0	1	1	1	0	1	1	1	1
	X_82_	0	1	1	1	0	1	1	1	1	1
	X_83_	0	1	1	1	1	1	0	1	1	1
	X_84_	1	1	1	1	1	1	1	1	1	1
X_9_	X_91_	1	1	1	1	1	1	1	1	1	1
	X_92_	1	1	1	1	1	1	1	1	1	1
	X_93_	0	1	1	0	0	1	0	0	1	0
	X_94_	1	1	1	1	1	1	1	1	0	1

**Table 6:** PMC-Index of 10 representative policies.

Item	P1	P2	P3	P4	P5	P6	P7	P8	P9	P10	Average
X_1_	1.00	1.00	1.00	0.83	1.00	1.00	1.00	1.00	1.00	1.00	0.98
X_2_	0.67	0.67	0.67	0.33	0.67	0.67	0.67	0.33	0.67	0.33	0.57
X_3_	1.00	1.00	1.00	1.00	1.00	1.00	1.00	1.00	0.75	1.00	0.98
X_4_	0.80	1.00	0.60	0.80	0.40	0.60	0.60	0.80	1.00	1.00	0.76
X_5_	0.80	0.80	0.80	1.00	0.80	0.80	0.80	0.80	0.80	0.80	0.82
X_6_	0.83	0.83	0.67	1.00	0.67	0.67	0.50	1.00	0.67	0.83	0.77
X_7_	1.00	1.00	1.00	1.00	1.00	0.83	0.83	1.00	0.67	0.83	0.92
X_8_	0.50	0.75	1.00	1.00	0.75	0.75	0.75	1.00	1.00	1.00	0.85
X_9_	0.75	1.00	1.00	0.75	0.75	1.00	0.75	0.75	0.75	0.75	0.83
PMC–index	7.35	8.05	7.73	7.72	7.03	7.32	6.90	7.68	7.30	7.55	7.46
Rank	6	1	2	3	9	7	10	4	8	5	

**Policy Nature (X**_**1**_**):** The average value for Policy Nature (X_1_) is 0.98, indicating that nearly all selected smart aging care policies address elements related to prediction, guidance, support, regulation, description, and diagnosis. Only one policy lacks content related to “prediction,” showing a deficiency in forward-looking perspectives. The remaining policies cover all six aspects, demonstrating that the selected policies are relatively comprehensive and well-developed.

**Policy Timeliness (X**_**2**_**):** The average score for Policy Timeliness (X_2_) is 0.57, the lowest among all indicators. This indicates that most policies failed to integrate short-term, medium-term, and long-term objectives in a progressive manner. The majority focused solely on long-term goals, prioritizing the ultimate outcomes after policy implementation while neglecting short- and medium-term targets. There was a lack of consideration for continuously adjusting objectives based on evolving circumstances during implementation.

**Policy Domain (X**_**3**_**):** The average score for Policy Domain (X_3_) is 0.98, tying with Policy Nature (X_1_) as the highest-scoring indicator. This indicates that all 10 smart aging care policy texts encompassed the four domains of economy, healthcare, technology, and social services. Their content featured multidimensional, multilevel, and multi-domain coverage, reflecting how smart aging care promotes sustainable and healthy societal development across multiple dimensions.

**Policy Objects (X**_**4**_**):** The average score for Policy Objects (X_4_) is 0.76. Smart aging care involves diverse and multifaceted objects, including governments, enterprises and institutions, research institutes, communities, and families. The scoring results indicate that some policies fail to comprehensively consider all stakeholders involved in the implementation of smart aging care, focusing only on 2–3 subjects, particularly neglecting foundational entities like communities and families.

**Policy Tool (X**_**5**_**):** The average score for Policy Tool (X_5_) is 0.82. This indicates that most selected policies incorporate incentive-based, innovative, and collaborative approaches. However, they show slight deficiencies in market-oriented measures, failing to establish reasonable and effective market mechanisms for commercial products like smart aging care solutions and senior insurance. Regarding mandatory tools, most policies lack mandatory provisions, relying primarily on encouragement and incentives. This approach may create relative obstacles during the implementation of smart aging care policies.

**Policy Content (X**_**6**_**):** The average score for policy content (X_6_) is 0.77. Most smart aging care policy texts include planning for “smart health-oriented aging care,” “application in older adult care service scenarios,” “age-friendly renovations,” and “digital platform development.” However, they exhibit deficiencies in areas such as “training and education for the older adults” and “insurance for older adults.” If the fundamental capacity of older adults to independently use smart aging care products is not fostered at the source, and efforts remain solely focused on research, development, and production, how can the true objectives of smart aging care be achieved. As a foundational safeguard in smart aging care, “insurance for older adults” must be incorporated into policy formulation to ensure the effective implementation of smart aging care initiatives.

**Policy Objectives (X**_**7**_**):** The average score for Policy Objectives (X_7_) is 0.92, ranking second only to X_1_ and X_3_. This indicates that policies were formulated with diverse and comprehensive objectives, encompassing product innovation, technology application, model promotion, standard setting, improvement of older adult care service quality, and development of the silver economy industry.

**Policy Measures (X**_**8**_**):** The average score for Policy Measures (X_8_) is 0.85. This indicates that some smart aging care policy texts did not sufficiently consider backup resources such as legal safeguards, funding support, and talent development, which require strengthening.

**Policy Evaluation (X**_**9**_**):** The average score for Policy Evaluation (X_9_) is 0.83. This indicates that the selected smart aging care policy texts emphasize having sufficient basis, clear objectives, and scientific solutions. However, the planning of solutions remains unclear and lacks distinction between the short-term, medium-term, and long-term goals outlined in X_2_.

Generally, based on the PMC rating standards proposed by Estrada (15) and subsequent adjustments by related scholars (49), the PMC-Index can be categorized into five grades. As shown in Table 7, the index ranges from 0 to 9, with higher values indicating greater policy consistency. The grades are as follows: [0, 2) is considered Poor; [2, 4) is Fair; [4, 6) is Good; [6, 8) is Outstanding; and [8, 9] is Excellent. This classification system facilitates subsequent quantitative policy analysis and lays a solid foundation for policy optimization and adjustment. Based on the PMC index in Table 7, the average PMC score for the 10 smart aging care policy documents is 7.46, which falls within the “Outstanding” tier of the policy text classification. This indicates that the selected policy texts exhibit relatively high quality, comprehensive content, practical relevance, and thorough consideration of implementation outcomes.

**Table 7:** Policy sample rating based on the PMC index model.

Score range	[8, 9]	[6, 8)	[4, 6)	[2, 4)	[0, 2)
**Level**	Excellent	Outstanding	Good	Fair	Poor

The 10 smart aging care policies are ranked from highest to lowest scores as follows: P2 > P3 > P4 > P8 > P10 > P1 > P6 > P9 > P5 > P7. Among these, the PMC scores for policy texts P2, P3, P4, P8, and P10 exceed the average. Within the set of 10 policy texts, 3 national-level smart aging care policies scored above average, along with 1 provincial-level and 1 municipal-level policy. However, P2 received a “Excellent” rating, while the remaining 9 policies were rated “Outstanding.” This indicates that national-level smart aging care policy documents exhibit superior quality compared to provincial and municipal-level documents. Nevertheless, the PMC index scores of all 10 policy texts reflect that smart aging care policy documents maintain a generally high standard overall, demonstrating scientific rigor, rationality, and feasibility.

The average score for the 4 provincial and municipal policies was 7.23, while the 2 city-level policies averaged 7.43. This indicates that city-level smart aging care policy texts generally outperform provincial and municipal ones, with a stronger emphasis on practical implementation. The policy from Suzhou focuses on cultivating the silver consumption market, while Wuhan's policy strengthens the establishment of smart community aging care service scenarios, reflecting how local policies precisely respond to dual themes based on their specific contexts. Among these, Chongqing and Suzhou achieved relatively high scores for their smart aging care policy texts at 7.68 and 7.55 respectively. This underscores the need for tailored policy development based on local economic conditions and the aging demographics of the older populations. In contrast, Zhejiang and Shaanxi scored relatively lower at 6.90 and 7.03, indicating a need to strengthen their policy formulation by comprehensively considering provincial development and practical circumstances.

### 4.3 Analysis of debra chart

**Figure 4:**
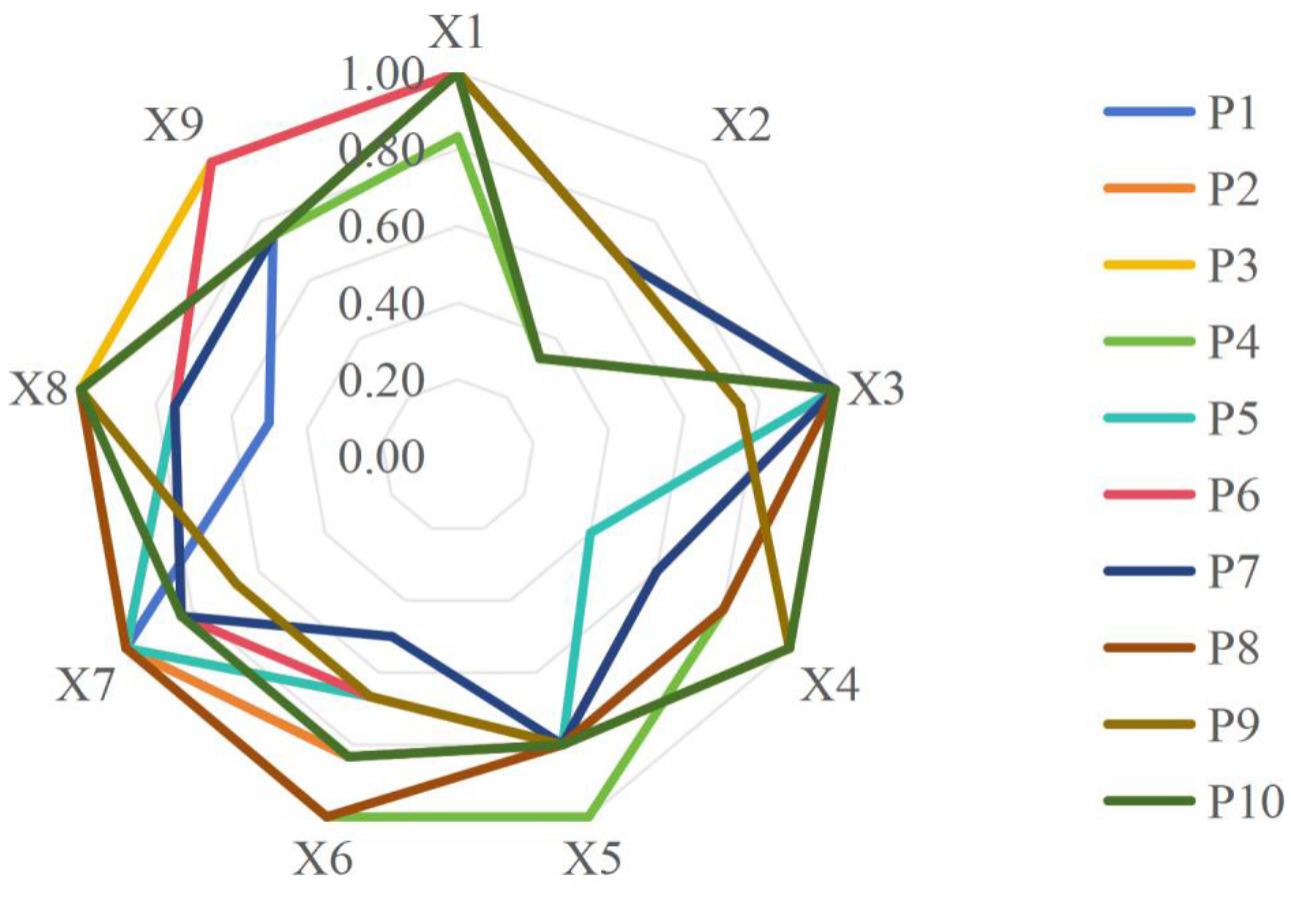
Debra chart of 10 representative policies. Note: The Smart aging Care Policy PMC-Index Radar Chart uses nine core primary variables as radial dimensions, with scores ranging from 0 to 1. A score closer to 1 indicates superior performance in that dimension.

To visually present the comprehensive performance and internal structural characteristics of various smart aging care policies, a multidimensional assessment of 10 representative policies was conducted based on the PMC-Index model, with debra chart created for visual analysis (Figure 4). The results reveal distinct variations in policy distribution across different variables, reflecting the diversity and degree of adjustment in current smart aging care policies regarding goal setting, content design, safeguarding measures, and tool selection.

Overall, smart aging care policies exhibit balanced and sound performance in domain coverage, nature positioning, and textual standardization, reflecting a strong policy foundation and systematic characteristics. However, significant variations exist in goal setting, content refinement, safeguard mechanisms, and tool combinations. This reflects both the flexible adjustments made by policies at different levels to adapt to local realities and specific needs, and suggests that future policy optimization could further enhance precision, operability, and coordination in these dimensions to improve overall policy effectiveness and implementation outcomes.

### 4.4 Analysis of PMC-Surface for 10 Representative Policies

**Figure 5:**
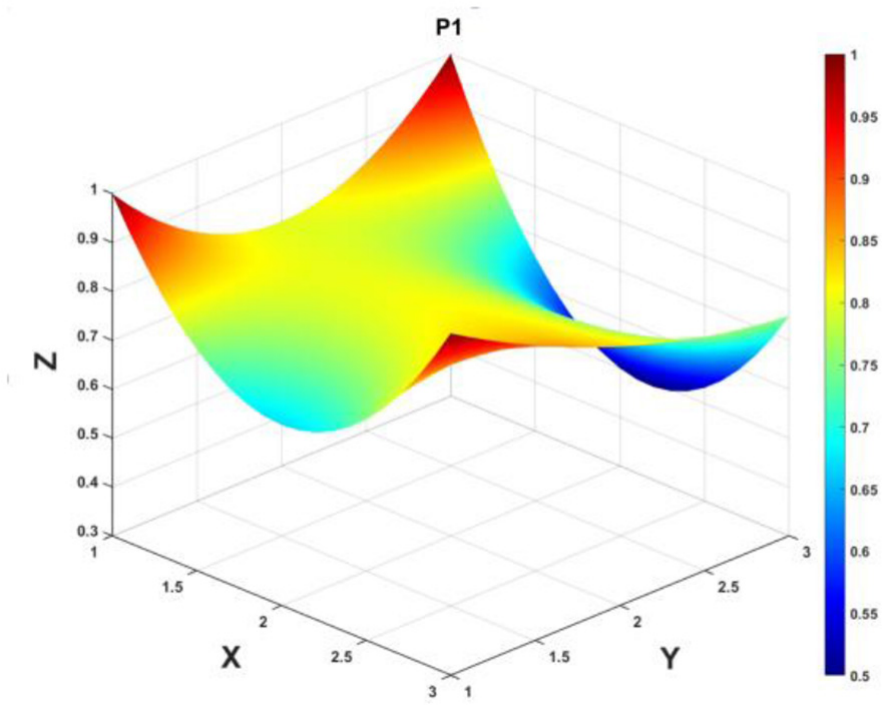
The PMC-Surface for P1.

**Figure 6:**
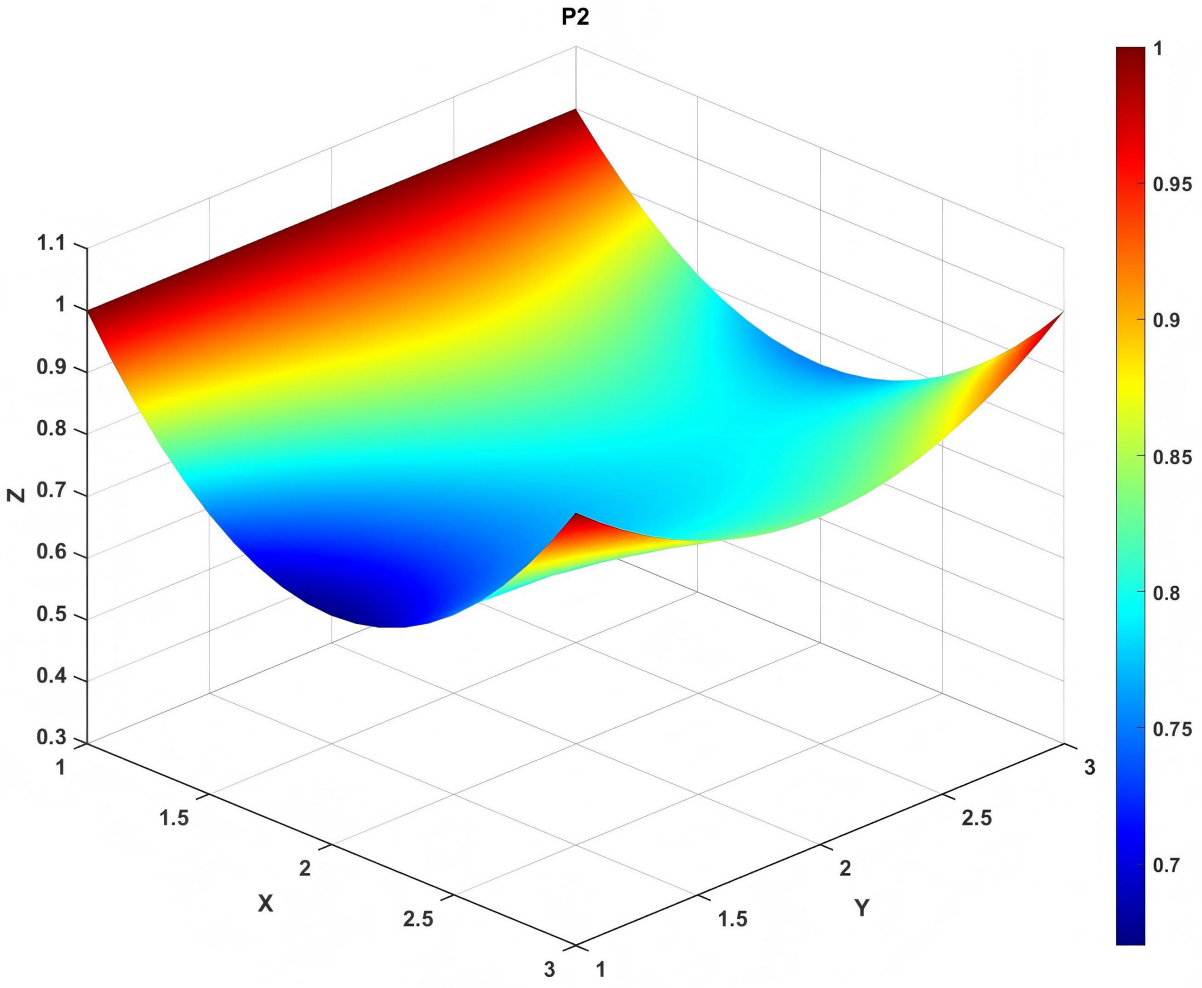
The PMC-Surface for P2.

**Figure 7:**
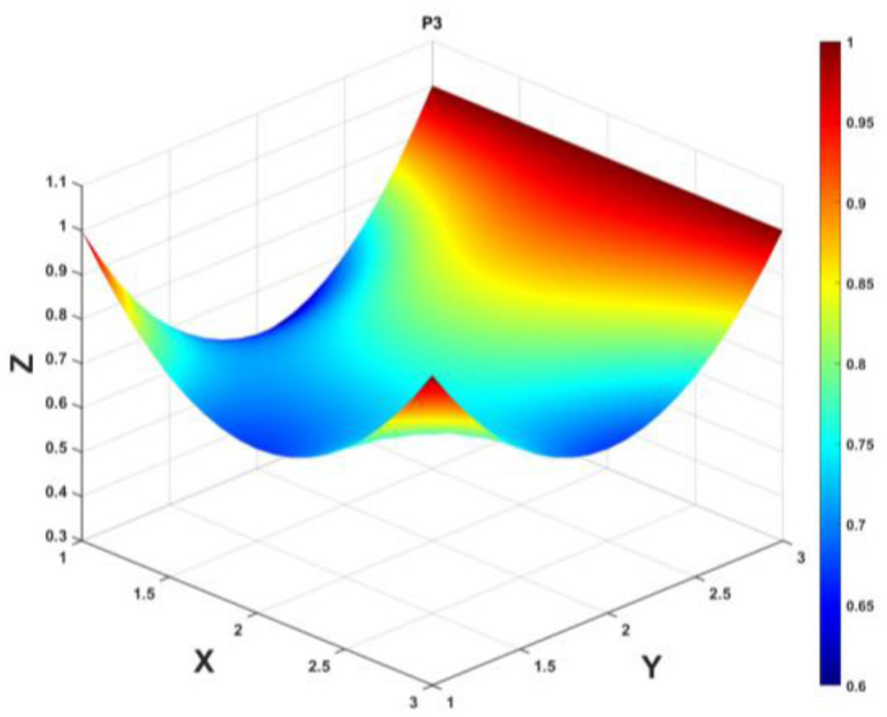
The PMC-Surface for P3.

**Figure 8:**
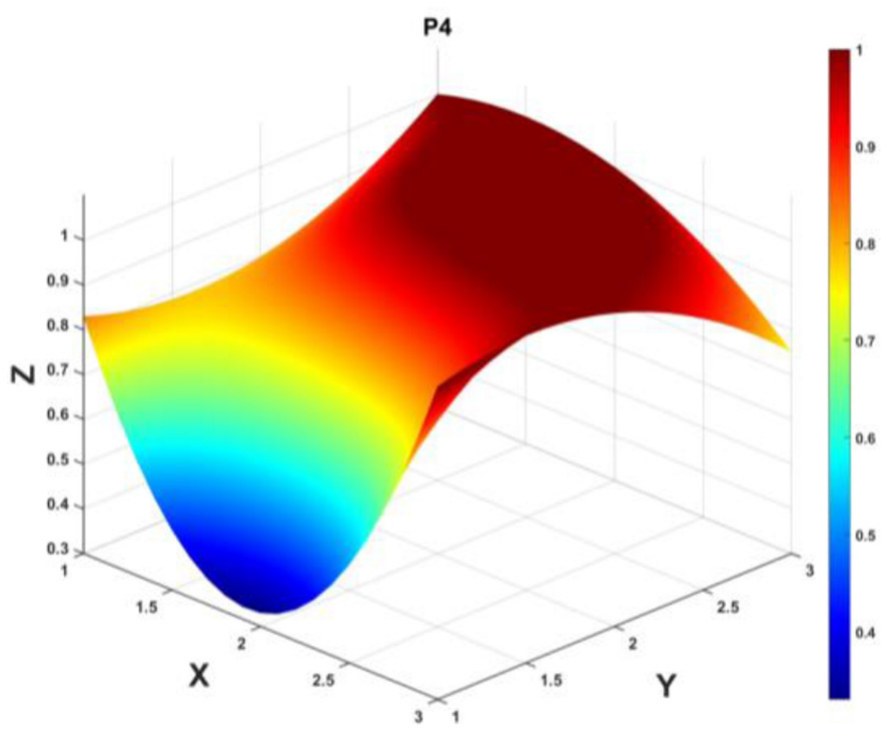
The PMC-Surface for P4.

**Figure 9:**
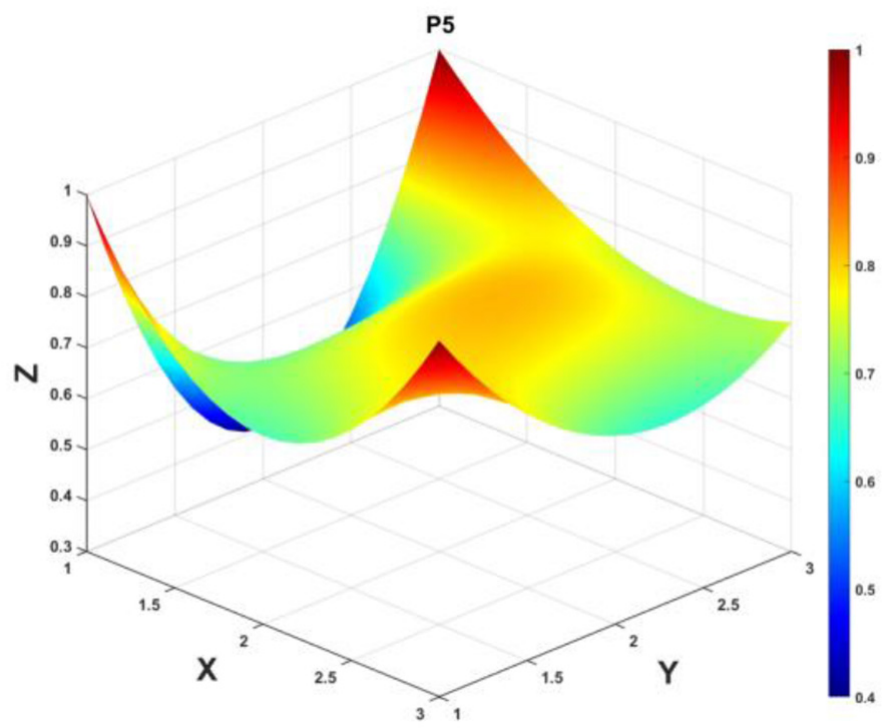
The PMC-Surface for P5.

**Figure 10:**
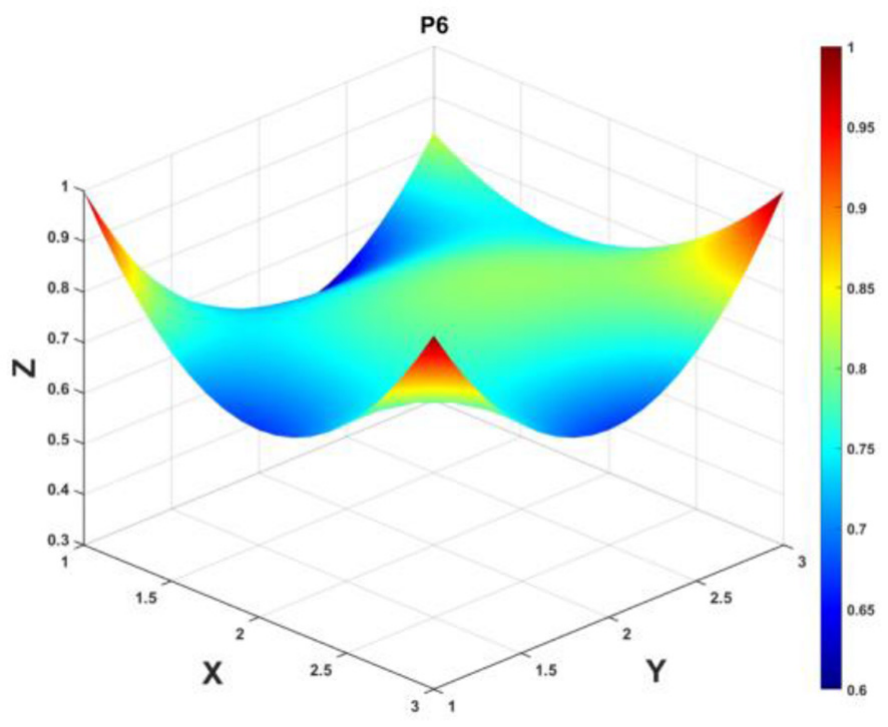
The PMC-Surface for P6.

**Figure 11:**
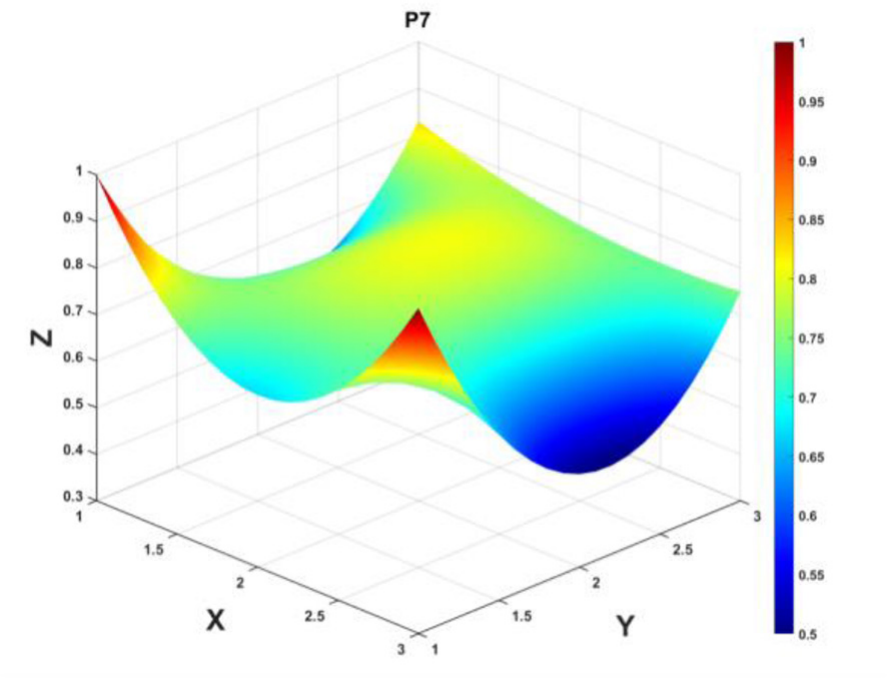
The PMC-Surface for P7.

**Figure 12:**
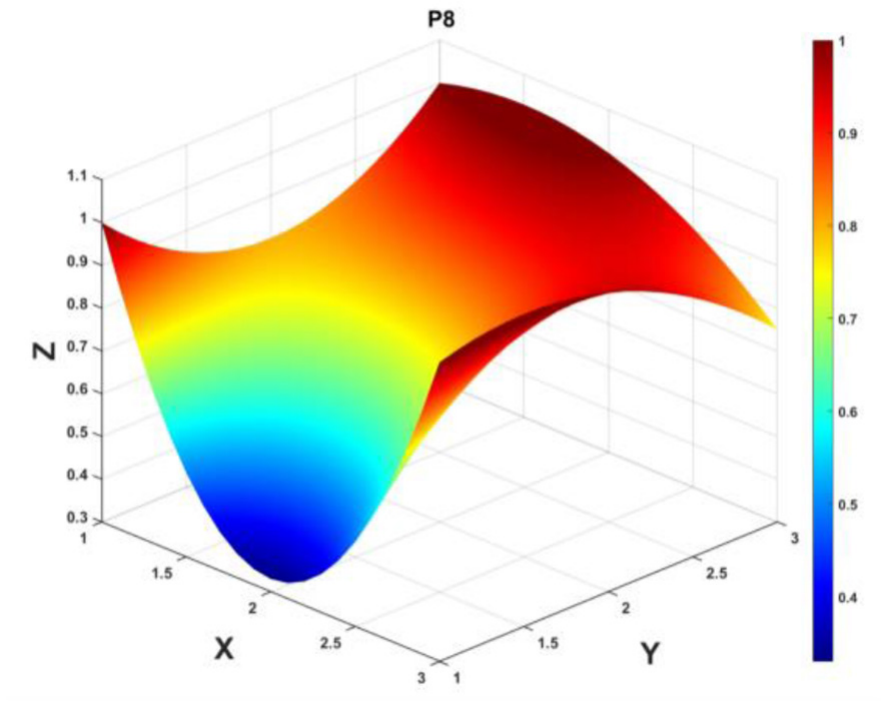
The PMC-Surface for P8.

**Figure 13:**
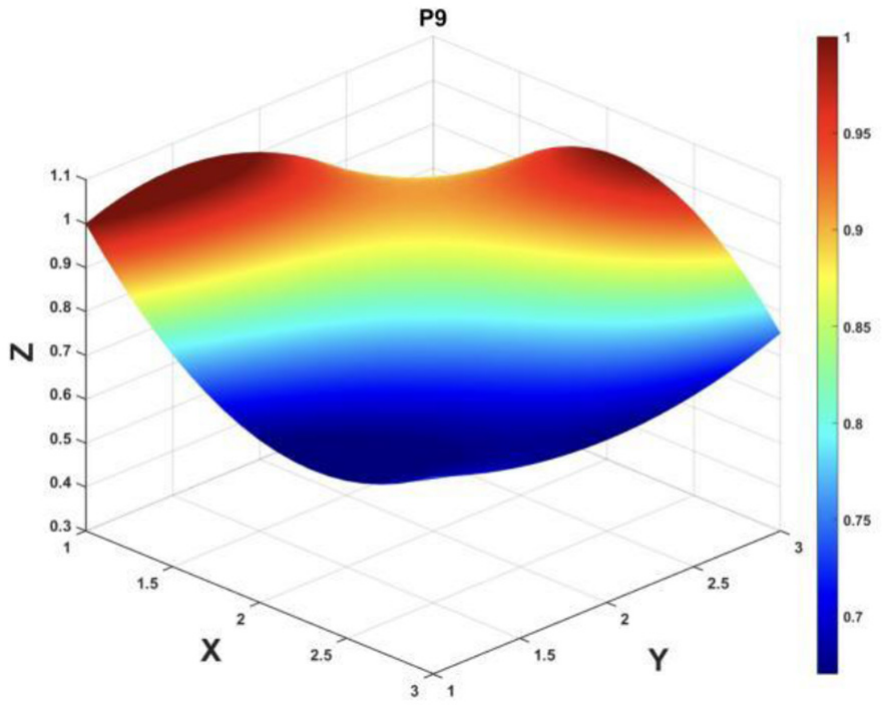
The PMC-Surface for P9.

**Figure 14:**
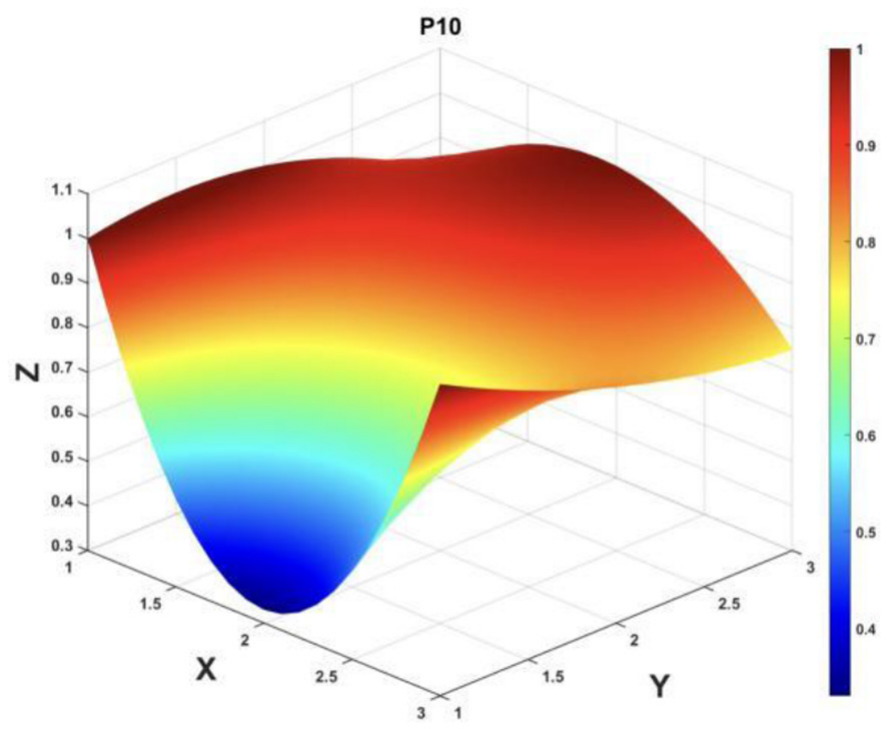
The PMC-Surface for P10. Figures 5-13 and this figure presents a three-dimensional surface plot of PMC-Surface. The X and Y axes correspond to a 3 × 3 matrix composed of nine primary variables, while the Z axis represents scores across dimensions (0–1). Colors range from red to blue, indicating decreasing scores from high to low. Convex surfaces denote high-value clusters, representing dominant dimensions, whereas concave surfaces indicate low-value clusters, representing weak dimensions.

To gain a deeper insight into the consistency level of smart aging care policies in terms of structure and content, and to reveal the performance differences across multiple dimensions, this study integrates the standardized scores of nine primary variables (X_1_-X_9_) into a 3 × 3 matrix based on the established PMC evaluation indicator system, as shown in Equation 5. Using this matrix as a foundation, MATLAB software is employed to generate a three-dimensional PMC-Surface diagram. In the Figures 4–13, the x-axis and y-axis correspond to the matrix row-column layout, while the z-axis represents the scores of each indicator. Surface colors transition from red to blue, indicating scores from high to low, thereby forming an intuitive spatial topological representation (47, 48). This visualization method not only presents the coordination and completeness of policies across nine policy dimensions through color block distribution and terrain undulations but also highlights strengths and weaknesses within policy structures. It provides spatial diagnostic evidence for subsequent targeted optimization. The following sections will systematically analyze the performance characteristics and overall consistency levels of ten smart aging care policies across variable groups using the PMC-Surface diagram (Figures 5–14).

**Policy P1**, “Implementation Plan for Effectively Addressing Older Adult Individuals' Difficulties in Using Smart Technologies,” scored 7.35 on the PMC-Index, ranking sixth among the ten policies and receiving an “Outstanding” rating. Issued by the General Office of the State Council, it aligns closely with China's strategic direction for digital inclusion of the older adults and proactive response to population aging. However, the policy scored only 0.5 points in the Policy Measures (X_8_) dimension, revealing a significant weakness. Particularly weak support in areas such as legal basis, funding, and talent development may hinder its implementation effectiveness. The PMC-Surface diagram reveals a generally flat surface, indicating a relatively balanced policy structure but lacking prominent peaks of strength. Future optimization should focus on enhancing the systematic nature and operability of the guarantee mechanism, further refining scenario-based content design, and enriching the policy toolkit to improve overall coordination and implementation effectiveness. The recommended optimization path is: *X*_8_→*X*_5_→*X*_6_→*X*_2_→*X*_3_→*X*_1_.

**Policy P2**, “Action Plan for the Development of the Smart Health and Older Adult Care Industry (2021–2025),” scored 8.05 in the PMC-Index evaluated this time, ranking first among the 10 policies with an “Outstanding” rating. Jointly issued by the Ministry of Industry and Information Technology and the Ministry of Civil Affairs, this policy serves as a specialized industrial blueprint for integrating the silver economy with digital technologies during the 14th Five-Year Plan period. It embodies a strategic orientation of empowering older adult care through industrial economics and driving services with novel intelligent age-friendly technologies. The PMC curve chart reveals a high and stable overall performance, indicating excellence and structural balance across most dimensions. It aligns particularly well with current development trends in product innovation, technology application, and silver economy growth. However, room for improvement exists in the dimensions of timeliness (X_2_) and measures (X_8_). Future refinements could focus on phased implementation planning, concretizing safeguards, and enhancing alignment with local practices to boost dynamic adaptability and implementation effectiveness. The recommended optimization path is: *X*_8_→*X*_2_.

**Policy P3**, “Work Plan for Promoting High-Quality Development of Digital Technologies Adapted for the Older Adults,” achieved a PMC-Index score of 7.73, ranking second in the evaluation with an “Outstanding” rating. Issued by the Ministry of Industry and Information Technology, this policy focuses on enhancing the quality and establishing a framework for digital technologies adapted for the older adults, reflecting a strategic orientation that integrates technological innovation with high-quality development (34). According to the PMC-Surface diagram analysis, the policy curve exhibits an overall gentle slope, aligning with the strategic direction of digital technology empowerment in older adult care services and demonstrating high consistency. However, the two dimensions of policy object (X_4_) and policy content (X_6_) exhibit pronounced concave tendencies, lacking multi-stakeholder and multi-dimensional considerations for the future development direction of smart aging care. It can comprehensively utilize multiple entities, leverage the foundational role of family and community-based smart aging care, and increase basic digital training and insurance coverage for the older adults. To further enhance its structural integrity and implementation effectiveness, the following optimization path is recommended: *X*_4_→*X*_6_→*X*_2_→*X*_8_→*X*_5_→*X*_3_.

**Policy P4**, “Several Measures to Further Promote Older Adult Care Service Consumption and Enhance the Quality of Life for the Older Adults,” achieved a PMC-Index score of 7.72, ranking third and receiving an “Outstanding” evaluation grade. This policy focuses on incentivizing older adult care service consumption and enhancing service quality, aligning closely with national directives on the silver economy and consumption upgrades among the older adult. It reflects a policy evolution from “ensuring basic needs” to “enhancing quality.” The PMC-Surface diagram demonstrates significantly low scores in both policy nature (X_1_) and policy timeliness (X_2_) dimensions, with a concave surface prominence. This indicates the absence of clear future development planning and ambiguous alignment between short-term, medium-term, and long-term objectives. Future improvements could involve engaging diverse research institutions for technical services and product innovation. Recommended optimization path: *X*_2_→*X*_1_→*X*_4_→*X*_9_.

**Policy P5**, “Shaanxi Province Work Plan for Promoting High-Quality Development of Digital Technology Adapted for the Older Adults,” scored 7.03 on the PMC-Index, ranking 9th with an “Outstanding” evaluation grade. As a provincial-level specialized plan implementing national digital aging-friendly requirements, this policy demonstrates local adaptability and detailed implementation. However, there remains room for improvement in its systematic approach and level of support. The PMC-Surface image shows several distinct depressions, particularly in the policy measures, object, and tool dimensions, indicating weaknesses in funding and legal support, multi-stakeholder coordination, and innovative tool combinations. While policy objectives and content align with national directives, they fall short in specificity and differentiation, lacking skill training for older adult users, basic digital literacy instruction, and insurance coverage. Recommended optimization path: *X*_8_→*X*_4_→*X*_5_→*X*_2_→*X*_6_→*X*_9_.

**Policy P6**, “Shanghai Action Plan for Advancing Older Adult Care Technology Innovation (2024–2027),” achieved a PMC index score of 7.32, ranking 7th with an “Outstanding” evaluation grade. This policy focuses on regional aging care technology innovation and industrial clustering, reflecting the municipality's pioneering exploration under the dual contexts of “technology-driven aging care” and “urban digital transformation.” The surface visualization of policy content (X_6_) and objectives (X_7_) dimensions presents a convex red area, with scores of 0.67 and 0.83 respectively, reflecting their high alignment with Shanghai's positioning in scientific and technological innovation in areas such as smart health care for the older adults and technology applications. However, the policy measures (X_8_) and object (X_4_) dimensions show concave surfaces with scores of 0.75 and 0.60 respectively, indicating deficiencies in legal safeguards, financial support, and coverage of family-community entities. The curvature profile indicates that the policy demonstrates strong performance in the technology-driven dimension, while its foundational support framework requires further enhancement. Recommended optimization path: *X*_8_→*X*_2_→*X*_4_→*X*_5_→*X*_6_→*X*_7_.

**Policy P7**, “Zhejiang Province Special Action Plan for Promoting High-Quality Development of Digital Technology Adapted for the Older Adults: “Zhejiang Age-Friendly”,” achieved a PMC index score of 6.90. Although it ranked 10th among the ten policies, its overall quality remains relatively high, earning an “Outstanding” rating. Jointly issued by multiple departments in Zhejiang Province, this policy aims to advance the adaptation of digital technology for the older adult through a specialized branded initiative. The PMC-Surface exhibits multiple blue depressions, with the policy content (X_6_, 0.50) and subject matter (X_4_, 0.60) dimensions showing the lowest scores, forming distinct low-lying areas. This reflects their dual shortcomings of vague content and narrow subject coverage. The policy measures (X_8_), evaluation (X_9_), and tool (X_5_) dimensions also fell below average levels, with the curve showing a slight concavity. The overall surface exhibits gentle undulations without prominent red protrusions, indicating that the policy lacks dominant dimensions and systematic support is insufficient. Therefore, it is recommended to follow the optimization path: *X*_6_→*X*_4_→*X*_8_→*X*_9_→*X*_5_.

**Policy P8**, “The Implementation Plan for Promoting the Silver Economy and Enhancing the Wellbeing of the Older Adults in Chongqing Municipality”, scored 7.68 on the PMC Index, ranking 4th with an “Outstanding” rating. This plan closely aligns with the national “silver economy” strategy, focusing on dual objectives of industry cultivation and welfare enhancement. It demonstrates the municipality's systematic planning in advancing the coordinated development of older adult care services and related industries. The PMC-Surface diagram is overall full-bodied. It indicates that the policy is relatively well-developed in service scenario design, target system construction, and supporting mechanism arrangements, aligning closely with the current national orientation of promoting innovation and high-quality development in the older adult care service sector. However, the policy timeliness (X_2_) score is relatively low, indicating weaker planning for short-term, medium-term, and long-term phases. The policy evaluation (X_9_) score also has room for improvement, particularly in enhancing planning clarity and scientific rigor. The recommended optimization path is: *X*_2_→*X*_4_→*X*_9_→*X*_5_.

**Policy P9**, “Implementation Plan for Wuhan's AI-Assisted Older Adult Care Social Experiment,” achieved a PMC index score of 7.30, ranking 8th with an “Outstanding” evaluation grade. As a social experiment project integrating AI with aging care at the prefecture-level city, this plan demonstrates local government innovation in the cutting-edge field of smart aging care and aligns with the national strategy of leveraging technology to empower older adult care services. The PMC-Surface diagram reveals a pronounced protrusion in both the policy object and measures dimensions, indicating outstanding performance in defining multi-stakeholder responsibilities and establishing implementation safeguards, demonstrating strong coordination and support capabilities. However, it exhibits flat or concave profiles across multiple dimensions including policy duration, coverage scope, and objectives. Notably, X_3_ scores only 0.75, indicating limited depth in economic and technological domains; the policy objective (X_7_) falls significantly below average, reflecting insufficient systematic planning in areas like model promotion and standard formulation. Recommended optimization path: *X*_3_→*X*_7_→*X*_6_→*X*_9_.

**Policy P10**, “Notice on Several Measures to Promote High-Quality Development of Suzhou's Silver Economy,” achieved a PMC index score of 7.55, ranking 5th with an “Outstanding” evaluation grade. This policy closely aligns with the dual themes of silver economy and high-quality development, reflecting local proactive exploration in synergizing industrial cultivation with public welfare advancement. It aligns with the current national policy orientation of promoting innovation and upgrading within the silver economy. However, the curve exhibits a pronounced dip in the policy timeliness (X_2_) dimension, falling below the average score. This indicates significant shortcomings in the integration of short-term, medium-term, and long-term planning. The policy evaluation (X_9_) score also has room for improvement, particularly in enhancing planning clarity and the scientific rigor of implementation plans. The recommended optimization path is: *X*_2_→*X*_9_→*X*_5_→*X*_6_.

## 5 Discussion

This study explores its core findings through the PMC-Index analysis of ten representative policies on smart aging care in China.

In terms of policy text characteristics, a distinct technological orientation is evident, reflecting the core focus on technology-enabled empowerment and the upgrading of older adult care services. Simultaneously, the policies demonstrate prominent industrial linkage, explicitly proposing the cultivation of new business models within the silver economy. This finding is consistent with the conclusion drawn by Wang and Tan in his research on smart aging care policies, which posits a continuous enhancement in the integration between policy and industry (50). Evaluation results reveal certain shortcomings in policy implementation, including temporal coordination, coverage scope, tool combinations, safeguarding measures, and evaluation mechanisms, which constrain overall effectiveness and outcomes. Regional comparisons reveal distinct spatial heterogeneity in China's smart aging care policies: Eastern regions prioritize high-end technological research and development and industrial innovation to enhance competitiveness; Central regions balance technology application with universal service access, emphasizing the enhancement of basic service systems; Western regions primarily aim to expand coverage and strengthen foundational safeguards. These differences primarily stem from variations in regional economic development levels, scientific and technological foundations, aging rates, and the structure of aging care needs. However, the analysis in this study primarily relies on policy text content and has not incorporated dynamic validation of policy implementation outcomes. Additionally, regional disparities in policies may exert certain influence on the interpretative validity of research conclusions. Based on influencing factors such as regional economic disparities, technological advancement levels, and older adult care service demands, this study proposes seven targeted policy optimization pathways to provide reference for promoting the high-quality development of smart aging care nationwide.

1. Regarding policy timeliness, a mechanism for dynamic iteration and phased advancement should be established. Within the long-term operational framework for smart aging care services, it is advisable to draw on the temporal management experience of Singapore's Smart Nation 2.0 initiative, which employs a rolling-update approach. This strategy replaces traditional static policy development with a continuous feedback loop for policy iteration (51), ensuring dynamic calibration aligned with the lifecycle of technologies like AI. This establishes a tiered objective system comprising short-term breakthroughs, Medium-term refinements, and long-term planning. Short-term (1–2 years): Focus on urgent tasks like technology deployment and age-friendly renovations, setting clear deadlines and quantifiable standards. Medium-term (3–5 years): Keep pace with technological evolution by integrating AI large-model applications for older adult care and incentives for online consumption by seniors into the policy framework. Long-term (5+ years): Prioritize deep integration of smart aging care with the silver economy, establishing a sustainable development framework. A cross-departmental dynamic evaluation team should be established. By adopting the policy adaptability assessment methodology summarized by Deng (52), which incorporates digital technology iteration cycles and the evolving needs of the older adults, regular policy reviews and revisions should be conducted. Concurrently, the timeliness management capacity of provincial policies should be strengthened to address issues of lag. This approach provides a significant reference for transforming China's smart aging care policies from long-term planning toward real-time resilient governance.
2. Regarding policy object, expand coverage and establish a multi-stakeholder collaborative system. Drawing on the core logic of multi-stakeholder coordination in Germany's multi-generational collaborative older adult care model (53), build a full-chain collaborative system featuring government coordination, enterprise leadership, and community/family participation. Strengthen coverage for vulnerable groups by incorporating community aging care service providers, senior product enterprises, smart aging care platform operators, and rural older adult households into the core service scope, while clearly defining the responsibilities and boundaries of all parties. Establish a routine feedback mechanism for beneficiary needs. According to the definition by the National Center for Assisted Living (NCAL), assisted living services are grounded in a person-centered care philosophy aimed at fulfilling residents' individualized needs and preferences (54). This approach centers on meeting the core needs of the older adults. By conducting community surveys and organizing focus groups with senior citizens, we can accurately identify diverse client requirements. This ensures precise alignment between policy provision and client needs, thereby enhancing collaborative effectiveness.
3. Regarding policy tools, optimize portfolio structures and enhance targeted empowerment. China can draw lessons from the robustness logic employed by East Asian neighbors in digital governance. As revealed by Zhang et al. in their empirical study on global regulatory pathways for AI-based medical devices, Japan and South Korea tend to seek a balance between robustness and efficiency in their policy instrument governance, in contrast to China's current approach, which emphasizes a catch-up model driven by policy incentives for domestic technology research and development (55). While leveraging incentive tools to enhance technology deployment efficiency, introduce stricter robustness assessments and risk constraint mechanisms to ensure the safety and reliability of smart aging technologies in complex application scenarios. This forms a diversified toolkit combining government-mandated regulations with market incentives. Mandatory tools establish safety thresholds, such as setting technical standards and market norms for smart aging-friendly products; innovating market-based tools to stimulate vitality, such as establishing specialized product design subsidies and interest-subsidized loans; and enriching collaborative tools to promote coordinated development by building platforms for government, enterprises, and research institutions to connect. Concurrently, regional adaptability of these tools should be enhanced: prioritizing market-based tools in eastern regions while strengthening incentive support in central and western regions. This approach enables precise allocation of policy resources and enhances overall effectiveness.
4. In terms of policy content, advance precision alignment and address core deficiencies. Regarding balanced regional governance in smart aging care services, a study by Russell on the sustainability outcomes of Canada's rural age-friendly projects reveals significant asymmetries in resource endowments, such as infrastructure, human capital, and fiscal support between urban and rural areas. This asymmetry dictates that rural age-friendly initiatives cannot simply replicate urban models (56). Based on this, we can learn from their resilient adaptation strategies: in resource-rich urban areas, policies should prioritize building highly integrated smart platforms; whereas in resource-scarce, geographically dispersed rural areas, the focus should be on establishing community-centered, low-cost, high-contact age-friendly projects, leveraging telemedicine and social support networks to compensate for physical resource limitations. Eastern regions should expand high-end smart device research and development and smart wellness tourism; central regions should strengthen county-level smart aging care service centers and talent training; western regions should prioritize telemedicine and simple smart device promotion as essential safety nets. Concurrently, core deficiencies must be addressed by integrating digital literacy training for seniors into public services, refining standards for aging-friendly modifications of smart devices, establishing dedicated research and development programs for senior products, and enhancing industrial support for aging-friendly products through policy (54).
5. Regarding policy objectives, refine quantitative expressions and strengthen logical coherence. In managing smart aging care policy goals, China can draw on Australia's governance experience in establishing outcome-oriented monitoring systems, leveraging multi-source data integration to achieve dynamic assessments of policy effectiveness. Research by Inacio et al. indicates that Australia's Register of Aged Persons (ROSA) has successfully established a national data repository by integrating cross-departmental information. This system not only enables in-depth empirical assessments of individual characteristics, diverse needs, and health outcomes within aging care settings but also facilitates routine monitoring of service accessibility, quality and safety, and technological innovation, alongside benchmarking against foundational standards (57). Establishing a clear, measurable quantitative target system and strengthening logical connections between objectives transforms policy goals from isolated administrative directives into evidence-based performance loops. This ensures smart aging care policies can capture emerging needs of the older populations in real time, while continuous feedback from quantitative indicators drives iterative optimization of policy tools. Simultaneously, differentiated objectives were set based on regional development realities: eastern regions prioritized innovation-driven quality enhancement, while central and western regions focused on coverage expansion and foundational consolidation. This approach ensures objectives are both scientifically grounded and achievable, aligning with the conclusion proposed by Cao that precise targeting is crucial for policy implementation (58).
6. In terms of policy measures, establish a diversified system and enhance support effectiveness. China can draw on Sweden's experience in building an inclusive aging care security system. Tynkkynen's research indicates that the Swedish model carries low systemic risk, primarily because it achieved an earlier transition from fragmented interventions to systematic integration. Sweden legally defined local governments' primary responsibility in older adult care services, establishing a highly integrated, decentralized security system with clear delineation of authority and responsibility (59). China can establish a universal security framework with clear responsibilities and cross-regional coordination, deeply integrating digital tools with a highly resilient social security network. First, solidify the legal foundation by advancing legislation for smart aging care service management regulations, clarifying core legal principles such as data privacy boundaries and service quality oversight. Second, strengthen diversified investment mechanisms. Establish a multi-source investment model guided by central finances, supplemented by local resources, and involving social capital. Targeted policy support for central and western regions should be achieved through national-level special development funds. Next, optimize talent development pathways and deepen university-enterprise collaborative education models. Finally, build a nationwide integrated smart aging care data sharing platform. This platform should ensure information interoperability while safeguarding data security, providing robust support for scientific and precise policy decision-making.
7. In policy evaluation, establish closed-loop mechanisms and enhance scientific rigor. Bicket et al., through empirical research on the 2020 revision process of The Magenta Book guidelines, argued that evaluations of complex social systems should not be confined to linear causal reasoning. Instead, they should leverage interdisciplinary dialogue and multi-stakeholder consultation to identify dynamic feedback loops in policy implementation, thereby constructing an evaluation logic that involves diverse actors (60). Focus on policy text integrity and broaden the scope of evaluation entities by forming multi-stakeholder evaluation teams comprising government, research institutions, service providers, older populations, and enterprises. Secondly, transform evaluation functions from *post-hoc* reflection to real-time adjustments during implementation. Through continuous feedback from diverse stakeholders, identify risks of misalignment between technological iterations and older adult needs. Establish a rigid linkage mechanism between evaluation outcomes and policy revisions, thereby providing scientific evidence to optimize tools and precisely adjust smart aging care policies.

## 6 Conclusion

Against the dual backdrop of continuously deepening population aging and vigorous digital economic development, smart aging care has emerged as a pivotal instrument for addressing the challenges of an aging society and catalyzing new sources of economic growth (12). Anchored in this contemporary context, this study employs the PMC index model to conduct a systematic evaluation of 10 representative smart aging care policies. The analysis focuses on policy documents that were enacted between 2020 and 2025 and selected for their representativeness within the field.

Research findings indicate that China's smart aging care policy framework demonstrates sound design, clear objectives, and comprehensive coverage. Policy documents exhibit high overall quality, characterized by distinct features of technology-driven empowerment and industry synergy. However, current policies face significant shortcomings in timeliness, with most lacking a tiered approach linking short-term, medium-term, and long-term goals. This hinders dynamic adaptation to technological evolution and shifting market demands. Furthermore, structural imbalances persist in policy coverage, content refinement, tool combinations, and safeguard mechanisms. Particular attention is needed for foundational entities like communities and households, while market-based tools and legal support remain relatively weak, undermining policy coordination and implementation effectiveness. Building on this, proactive efforts should be made to promote international experience exchange and cross-regional cooperation, jointly advancing research and development innovation for smart aging care products and key technologies. Governments must further leverage their macro-level guidance and coordination roles to establish unified, collaborative smart aging care standards and service norms, providing robust support for policy implementation and the industry's healthy development.

From a theoretical perspective, the Smart Aging Care Policy PMC evaluation indicator system developed in this study overcomes the qualitative limitations of traditional policy text analysis. It achieves systematic quantitative measurement of multi-dimensional indicators including policy content, tools, timeliness, and safeguards, thereby providing an operational theoretical framework and methodological pathway for scientific evaluation and comparative research on smart aging care policies. From the practical perspective, this study provides the solid foundation for policy refinement and precise implementation through an in-depth evaluation of 10 representative policies. This article presents the following actionable optimization recommendations for policymakers. At the policy-making level, it is recommended to establish a dynamically adjusted “policy toolkit” list, categorizing incentive-based, mandatory, and market-oriented tools according to applicable scenarios, and formulating differentiated policies tailored to the varying needs of eastern, central, and western regions. Detailed implementation rules for region-specific policies should be introduced accordingly. At the policy implementation level, a cross-departmental policy coordination task force should be established to facilitate information sharing among departments such as civil affairs, health commission, and industry and information technology through the national integrated data platform. Additionally, grassroots policy implementation observation points should be set up to regularly collect practical feedback from communities, households, and older adult care institutions. At the policy evaluation level, it is recommended to incorporate policy implementation effectiveness into the performance assessment system of local governments, introduce third-party institutions for annual dynamic monitoring, and publish professional guidance documents on the effectiveness of smart aging care policy implementation, while publicly disclosing policy optimization progress to society. The aforementioned recommendations aim to facilitate the transition of smart aging care policies from “policy document completeness” to “practical implementation”, thereby providing robust institutional safeguards for the high-quality development of the silver economy.

The present study has the following limitations. First, the PMC-Index model exclusively focuses on quantitative analysis of policy texts, employing a cross-sectional analytical design that conducts static evaluations of policy documents at specific time points. This approach fails to reveal the dynamic evolutionary logic underlying policy adjustments and optimizations, thereby limiting its capacity to infer causal relationships. Secondly, in terms of sample coverage, not all provinces and regions were included, indicating room for improvement in sample completeness. Third, regarding variable selection, while the PMC-Index model encompasses multidimensional characteristics of policy texts, it fails to incorporate external variables that may influence policy implementation outcomes, particularly lacking cross-validation for health outcomes among older adult populations (i.e., life expectancy). To address the aforementioned limitations, future research may be further expanded in the following directions. Firstly, longitudinal policy evaluation studies should be conducted by employing a diachronic analytical framework to track the adjustment trajectories of individual policies or policy clusters across different time periods. This approach reveals the dynamic logic and iterative patterns of policy evolution, thereby addressing the limitations of causal inference inherent in cross-sectional designs. Secondly, expand the sample of grassroots policies by incorporating prefecture-level cities, counties, and townships into the evaluation scope. Conduct stratified and categorized assessments that account for urban-rural disparities, regional gradients, and differences in policy types, thereby enhancing the generalizability and practical value of the research conclusions. Thirdly, we introduce health outcome indicators for the older adult population (e.g. life expectancy) as validation criteria for policy implementation effectiveness. By establishing a causal chain model linking policy quality to implementation outcomes, we develop a dynamic evaluation framework (61) to systematically examine the genuine impact of smart aging care policies on the health and wellbeing of the older adult population, thereby overcoming the current research limitations that remain confined to static textual analysis of policies. Fourthly, broaden the perspective of transnational comparative studies by selecting countries with mature models of smart aging care and silver economy development for comparative analysis of policy texts. This approach aims to delve into national development disparities and institutional learning experiences, providing international references for China's precise policy adjustments and sustainable implementation. Through the above research, we can build a more systematic, dynamic and international perspective of smart pension policy evaluation system, which will provide more solid theoretical support and practical guidance for promoting the high-quality development of China's smart pension industry and promoting the sustainable prosperity of silver economy.

## Data Availability

The original contributions presented in the study are included in the article/supplementary material, further inquiries can be directed to the corresponding authors.
